# A universal tool for predicting differentially active features in single-cell and spatial genomics data

**DOI:** 10.1038/s41598-023-38965-2

**Published:** 2023-07-22

**Authors:** Alexis Vandenbon, Diego Diez

**Affiliations:** 1 Institute for Life and Medical Sciences, Kyoto University, 53 Shougoin Kawahara-cho, Sakyo-ku, Kyoto, 606-8507 Japan; 2 Institute for Liberal Arts and Sciences, Kyoto University, Yoshidanihonmatsu-cho, Sakyo-ku, Kyoto, 606-8501 Japan; 3 Immunology Frontier Research Center, Osaka University, 3-1, Yamada-oka, Suita, Osaka 565-0871 Japan; 4 Open and Transdisciplinary Research Institute (OTRI), Osaka University, 1-1, Yamada-oka, Suita, Osaka 565-0871 Japan

**Keywords:** Gene expression analysis, Data mining, Machine learning, Computational biology and bioinformatics, Software

## Abstract

With the growing complexity of single-cell and spatial genomics data, there is an increasing importance of unbiased and efficient exploratory data analysis tools. One common exploratory data analysis step is the prediction of genes with different levels of activity in a subset of cells or locations inside a tissue. We previously developed singleCellHaystack, a method for predicting differentially expressed genes from single-cell transcriptome data, without relying on comparisons between clusters of cells. Here we present an update to singleCellHaystack, which is now a universally applicable method for predicting differentially active features: (1) singleCellHaystack now accepts continuous features that can be RNA or protein expression, chromatin accessibility or module scores from single-cell, spatial and even bulk genomics data, and (2) it can handle 1D trajectories, 2-3D spatial coordinates, as well as higher-dimensional latent spaces as input coordinates. Performance has been drastically improved, with up to ten times reduction in computational time and scalability to millions of cells, making singleCellHaystack a suitable tool for exploratory analysis of atlas level datasets. singleCellHaystack is available as packages in both R and Python.

## Introduction

Recent advances in single-cell and spatial omics technologies allow researchers to obtain abundance measures of transcripts and proteins, or the accessibility of genomic regions at single-cell resolution. These technologies present an unprecedented view of the heterogeneity in cell populations and their spatial distributions within tissues. However, they are also accompanied by new challenges in data analysis.

A fundamental step in exploring single-cell transcriptomics data is predicting genes that have different levels of expression in one subset of cells compared to others. Such genes are often referred to as differentially expressed genes (DEGs). Similarly, in spatial transcriptomics data, spatially variable genes (SVGs) are genes with altered expression in part of a tissue. In single-cell ATAC (scATAC-seq) data, differentially accessible regions (DARs) are genomic regions which have a higher accessibility in one group of cells compared to others. In this paper, we will use the term differentially active features (DAFs) to refer to any feature or set of features with differential levels of activity (including DEGs, SVGs and DARs) within an input space, be it the 2D or 3D space within a tissue or latent spaces (such as principal components, tSNE or UMAP) of any dimension.

The majority of single-cell DEG prediction approaches are based on two steps: (1) clustering of cells by similarity, and (2) applying statistical tests between clusters to identify DEGs^1–7^. However, benchmark studies have reported that DEG prediction approaches for bulk RNA-seq do not perform worse than methods designed specifically for single-cell RNA-seq (scRNA-seq), and that the agreement between single-cell DEG prediction approaches is low^8,9^. Because the number of clusters tends to be large, a common approach is to compare the cells in each cluster against all other cells, which restricts DEGs that can be detected to genes with high (or low) expression in a single cluster. For the prediction of SVGs, methods have been developed that directly employ the spatial coordinates of cells (or spots or pucks) to detect genes that have non-random distributions of expression in the 2D (or 3D) space of the tissue^10–17^. However, most existing methods do not scale well with large datasets, suffer from prohibitively long runtimes, and are limited in the spatial patterns that they can detect in practice. The development of more flexible approaches for discovering complex differential expression patterns is one of the grand challenges of this field^18^.

We recently developed singleCellHaystack, a method that predicts DEGs based on the distribution of cells in which they are active within an input space^19^. Our method does not rely on comparisons between clusters of cells and is applicable to both scRNA-seq and spatial transcriptomics data. Although singleCellHaystack was superior to other methods in our extensive comparison^19^, a limitation of the implementation was that it used a hard threshold for defining genes as being either detected or not detected in each cell. Treating detection in a binary way ignores the magnitude of gene expression differences, which might miss some differential expression patterns. Furthermore, singleCellHaystack was not able to handle sparse matrices, limiting its applicability to the ever-increasing dataset sizes.

Here, we present a new approach which addresses the above limitations and features several critical improvements. First, our new method uses continuous activity levels, enabling the identification of changes in any kind of features (e.g., RNA or protein concentrations, chromatin accessibility of genomic loci, etc.). Second, it uses cross-validation for choosing a suitable flexibility of splines during its modeling steps. Third, the computational time has been drastically reduced by incorporating several engineering improvements to the base code, including the use of sparse matrices. Finally, a Python implementation has been developed which enables the efficient application of singleCellHaystack to atlas level datasets with millions of cells. These improvements, together with the fact that it does not make strong assumptions about the statistical distribution of the input data, make singleCellHaystack applicable to a wide range of data types. In this manuscript we describe applications to single-cell transcriptomics, spatial transcriptomics (Visium, Slide-seqV2, HDST, and MERFISH), scATAC-seq, CITE-seq, and a large collection of bulk RNA-seq samples^20–24^. Moreover, our approach can also be used on sets of genes (e.g., genes sharing a common annotation) for predicting differential activities of biological pathways in single-cell and spatial transcriptomics data, and to identify DEGs along trajectories. Together, these results illustrate the usefulness of singleCellHaystack for exploring complex biological datasets. The new singleCellHaystack method is implemented as an R package and is available from CRAN (version 1.0.0 and higher) and GitHub, and as a Python package available from PyPI (version 0.0.5) and GitHub.

## Results

### Introduction to the singleCellHaystack method

**Figure 1:**
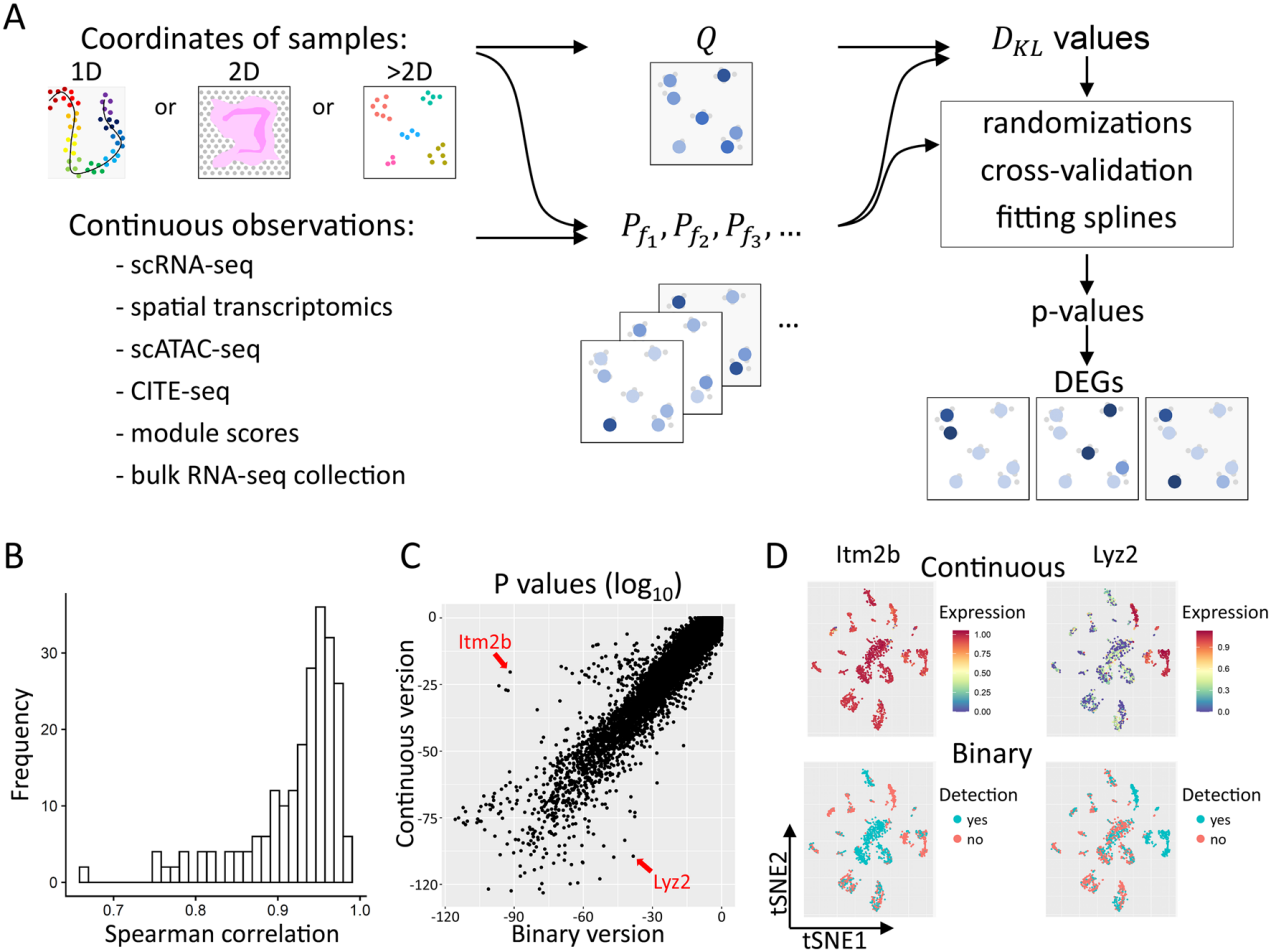
Concept of the new approach of singleCellHaystack and comparison with the previous binary approach. **(A)** A schematic overview of the new singleCellHaystack method. **(B)** Histogram of the Spearman correlation values between p-values estimated by the binary and the continuous versions of singleCellHaystack on 119 scRNA-seq datasets. **(C)** Scatter plot of p-values (log_10_) estimated using the binary (X axis) and continuous (Y axis) versions of singleCellHaystack on the Tabula Muris lung dataset. Two genes with large discrepancies in p-values are indicated. **(D)** tSNE plots showing the genes indicated in **(C)**. The top two plots show the normalized expression values used by the continuous version. The bottom two plots show the binarized detection levels as used by the binary version of singleCellHaystack.

Figure 1A shows a summary of the singleCellHaystack approach (version 1.0.0; see Methods for a more detailed description). In brief, singleCellHaystack requires two types of input data: (1) the coordinates of the samples (e.g., cells, spots, pucks, etc.) inside a space, which could be a 1D trajectory (e.g., pseudotime), 2D or 3D spatial coordinates, or a latent space such as principal components (PCs) or latent spaces returned by batch correction methods (such as Harmony, Scanorama, and scVI)^25–27^, and (2) a matrix of numerical values reflecting the activities of features in each sample (activity matrix). These typically are estimates of the concentrations of RNAs or proteins but can also be scores reflecting the accessibility of genomic regions or the average expression of sets of genes that share common functional annotations. First, singleCellHaystack estimates $Q$, the distribution of samples inside the input space. It does so by measuring the local density of samples around a set of grid points. Next, for each feature $f$ it estimates ${P}_{f}$, the distribution of the activity of $f$ inside the space by weighting the density of samples around each grid point by the activity of feature $f$. The difference between each ${P}_{f}$ and the reference distribution $Q$ is measured by using the Kullback–Leibler divergence ${D}_{KL}$^19^. For each ${D}_{KL}$ value a p-value is estimated using randomization of the activity matrix, and features with low p-values are regarded as DAFs. Because singleCellHaystack does not make comparisons between clusters, it allows detecting more complex patterns of differential activity.

### Comparison between singleCellHaystack’s binary and continuous methods

Our previous method (available in singleCellHaystack version 0.3.2) treated the expression of genes as binary (i.e. either expressed or not) using a hard threshold^19^. This new method (found in singleCellHaystack version 1.0.0 and higher) uses continuous values, reflecting the magnitude of the activity of each feature around each grid point, allowing it to identify more nuanced patterns of expression, and extending the application to features other than gene expression levels. To investigate differences in DEGs returned by the new continuous version and the original binary version of singleCellHaystack, we applied both versions to 119 scRNA-seq datasets of Tabula Muris and Mouse Cell Atlas^28,29^. Although both methods returned generally consistent results (the average Spearman correlation between log p-values of the binary and the continuous version was 0.92; Fig. 1B), in each dataset we also observed large discrepancies caused by the usage of the hard threshold in the binary version. We found that the binary version tends to emphasize highly-expressed genes with only small variations in expression (Supplementary Section “Comprehensive comparison of the binary and continuous versions of singleCellHaystack”, Supplementary Fig. S1). For example, both versions returned highly consistent results on the Tabula Muris lung tissue dataset (Spearman correlation 0.95; Fig. 1C), but several genes were judged to have differential expression by one version and not by the other. Two examples are *Itm2b* and *Lyz2* (Fig. 1C,D). *Itm2b* has stable expression levels across the cell clusters in the dataset (Fig. 1D top left plot). In contrast, *Lyz2* has high expression in a few subsets of cells, with lower expression in most others (Fig. 1D top right plot). In the binary version, the relatively small differences in expression of *Itm2b* become exaggerated because of the use of a hard threshold (Fig. 1D bottom left plot). On the other hand, for *Lyz2* the binary version dilutes the differential expression pattern because some cells with low expression also exceed the hard threshold (Fig. 1D bottom right plot). This leads to *Itm2b* being regarded as a top-scoring DEG by the binary version but not by the continuous version (ranked 42nd by the binary version; 2872nd by the continuous version), and the opposite result for *Lyz2* (ranked 1014th by the binary version; 42nd by the continuous version). Other examples are shown in Supplementary Fig. S2. To evaluate the robustness of both versions w.r.t. the input space used, we applied them to the Tabula Muris lung tissue dataset using different input spaces (the first 5, 20 or 50 PCs; Supplementary Fig. S3). Both versions returned in general robust results; top-scoring DEGs tend to remain top-scoring DEGs regardless of the input space used. However, naturally, the first 50 PCs contain information that is missing from the first 5 or the first 20 PCs, resulting in some discrepancies.

To evaluate the robustness of singleCellHaystack w.r.t. sample sizes, we applied it to down-sampled datasets and compared results with those obtained on the full datasets (Supplementary section “Dependency of singleCellHaystack on sample sizes” and Supplementary Fig. S4). As expected, the consistency of results decreased with decreasing samples sizes, but singleCellHaystack returned more robust results than the Wilcoxon rank sum test implemented in Seurat’s FindAllMarkers function.

In addition to the changes to the ${D}_{KL}$ computation, we made improvements to other parts of the implementation, including efficient use of sparse matrices, that resulted in shorter runtimes (Supplementary Fig. S5). The continuous version was faster than the binary version on all 119 Tabula Muris and Mouse Cell Atlas scRNA-seq datasets, with an average 37.8% reduction in runtime. In addition, we implemented singleCellHaystack in Python (https://github.com/ddiez/singleCellHaystack-py) enabling broader usability and the application to very large datasets. To show this we applied the Python version to a scRNA-seq dataset with 4 million cells from human fetal tissues^30^ (Supplementary Fig. 6A,B). This analysis took around 125 min to finish on a Dell Precision 7920 workstation with 26 cores and 500 GB of physical memory indicating that singleCellHaystack scales to atlas level datasets with millions of cells. To compare the performance of the Python and R implementations, we ran both pipelines in the same workstation on subsamples of the 4 M dataset. The results shown in Supplementary Fig. 6C indicate that the Python implementation computational time grows more slowly. Indeed, we were unable to run the full dataset in R. For datasets with up to 100 k cells, both implementations finish within 10 min. For larger datasets, using the Python implementation is recommended.

### Application to spatial transcriptomics data and comparison with existing methods

**Figure 2:**
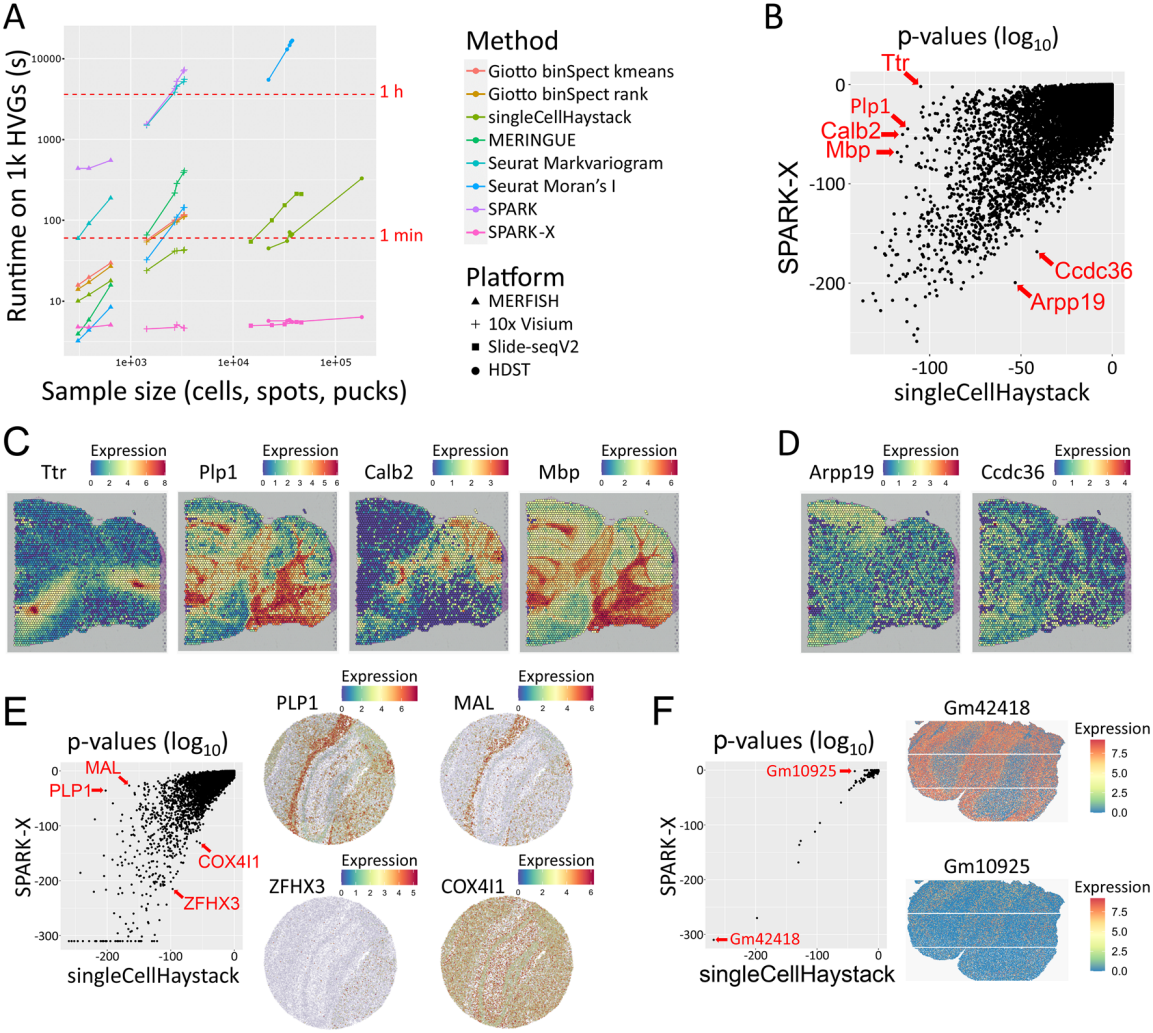
Applications to spatial transcriptomics data. **(A)** Comparison of runtime of several SVG prediction methods applied on 1000 HVGs of datasets of different platforms. For fair comparison, SPARK and SPARK-X were run on 1 core. Applications which failed to return results are not shown. **(B–F)** Example comparisons of the results of singleCellHaystack and SPARK-X. **(B–D)** Example application to mouse posterior brain (10x Visium, dataset “posterior1”). A scatterplot of p-values (log_10_) is shown **(B)** with indicated SVGs. Expression patterns in the mouse posterior brain are shown for high-scoring SVGs according to singleCellHaystack **(C)** and according to SPARK-X **(D)**. **(E,F)** Example applications on mouse hippocampus (Slide-seqV2) **(E)** and mouse olfactory bulb (HDST) **(F)**, showing a scatterplot of p-values (log_10_) with indicated SVGs (left side), and the expression patterns inside the tissue (right side).

We applied several SVG prediction methods on spatial transcriptomics data of several platforms (MERFISH, 10x Visium, Slide-seqV2, and HDST; see Table 1). The methods compared were singleCellHaystack, SPARK, SPARK-X, Seurat’s FindSpatiallyVariableFeatures using the Moran’s I and mark variogram approaches, MERINGUE, and Giotto’s binSpect using the kmeans and the rank approaches^12,15–17,19,31,32^. We first ran all methods on the top 1000 highly variable genes (HVGs) in each dataset and recorded their runtimes. Unfortunately, most methods do not scale well with increasing dataset size (Fig. 2A) or failed to run on the larger datasets. SPARK-X was in general the fastest method, followed by singleCellHaystack. Runtimes of singleCellHaystack are not solely a function of the number of spots (or cells, pucks) in the data, but also of the sparsity of the data. Because of this, runs on HDST datasets (which have a higher fraction of zeroes) took less time than runs on Slide-seqV2 datasets of similar sizes. To evaluate the consistency between singleCellHaystack and the other methods, we calculated the Spearman’s rank correlation between their p-values or scores on each dataset. Supplementary Fig. S7 shows the average correlation between the methods on the MERFISH and Visium datasets. singleCellHaystack and the other methods generally returned consistent results (typical correlations in the range 0.70 to 0.95). Larger discrepancies were seen for Markvariogram and SPARK-X on MERFISH data and for SPARK on Visium data.

**Table 1:** Overview of spatial transcriptomics datasets.

ID	Name	Platform	Species	Tissue/origin	References
1	Anterior1	Visium	Mouse	Brain	10x Genomics
2	Anterior2	Visium	Mouse	Brain	10x Genomics
3	Posterior1	Visium	Mouse	Brain	10x Genomics
4	Posterior2	Visium	Mouse	Brain	10x Genomics
5	Kidney	Visium	Mouse	Kidney	10x Genomics
6	Stickels_hippo1	Slide-seqV2	Mouse	Hippocampus	^20^
7	Stickels_hippo2	Slide-seqV2	Mouse	Hippocampus	^20^
8	Stickels_embryo	Slide-seqV2	Mouse	Embryo	^20^
9	Stickels_olfactory	Slide-seqV2	Mouse	Olfactory bulb	^20^
10	Stickels_cortex	Slide-seqV2	Mouse	Cortex	^20^
11	Vickovic_CN13_D2	HDST	Mouse	Olfactory bulb	^21^
15	Vickovic_CN24_D1	HDST	Mouse	Olfactory bulb	^21^
16	Vickovic_CN24_E1	HDST	Mouse	Olfactory bulb	^21^
12	Vickovic_CN21_C1	HDST	Human	Breast cancer	^21^
13	Vickovic_CN21_D1	HDST	Human	Breast cancer	^21^
14	Vickovic_CN21_E2	HDST	Human	Breast cancer	^21^
17	Xia_B1	MERFISH	Human	Osteosarcoma	^22^
18	Xia_B2	MERFISH	Human	Osteosarcoma	^22^
19	Xia_B3	MERFISH	Human	Osteosarcoma	^22^

For a genome-wide comparison on all genes of each dataset, we restricted ourselves to the two fastest methods (singleCellHaystack and SPARK-X). We applied both methods to each dataset and plotted the returned p-values (log_10_ values). We evaluated the differences in the mouse brain (10x Visium posterior1 slice; Fig. 2B,C), mouse hippocampus (Slide-seqV2; Fig. 2E) and mouse olfactory bulb (HDST; Fig. 2F). We further evaluated the differences in several other datasets using 10x Visium, Slide-seqV2, HDST and MERFISH technologies (Supplementary Figs. S8-S11). In several datasets, we observed that singleCellHaystack was able to pick up clear SVGs which were missed by SPARK-X. For example, in the mouse posterior brain 10x Visium dataset, top-scoring SVGs by singleCellHaystack included *Mbp*, *Calb2*, *Plp1*, and *Ttr* (Fig. 2B), all of which show clear spatially differential expression patters (Fig. 2C), yet were not among top-scoring SVGs predicted by SPARK-X. A striking example is the highly concentrated expression of *Ttr* (transthyretin) in 2 locations in the posterior brain. singleCellHaystack regards *Ttr* as a top-scoring SVG (p-value 1.1 × 10^–105^; ranked 58th out of 16,596 genes), but SPARK-X does not (p-value 0.0087; ranked 11,881st). In contrast, genes that are top-scoring according to SPARK-X but not singleCellHaystack are rare. Two such genes are Arpp19 (ranked 18th by SPARK-X; 983rd by singleCellHaystack) and Ccdc36 (ranked 73rd by SPARK-X; 1585th by singleCellHaystack) in the same posterior brain dataset (Fig. 2B). Although both genes exhibit some spatial variation of expression (Fig. 2D), the spatial pattern is visually less obvious than that of *Ttr* and other genes which have high expression concentrated in particular substructures of the tissue, but are missed by SPARK-X. Similar results were seen in other datasets from posterior brain, anterior brain, and kidney (Supplementary Fig. S8).

In Slide-seqV2 datasets, too, top-scoring SVGs picked up by singleCellHaystack but missed by SPARK-X were relatively common. For example, in the mouse hippocampus sample, several genes were top-ranking SVGs according to singleCellHaystack but not SPARK-X, including *Plp1* (ranked 20th by singleCellHaystack vs 1139th by SPARK-X) and *Mal* (ranked 55th by singleCellHaystack vs 1543rd by SPARK-X), all showing strong spatial expression patterns (Fig. 2E, Supplementary Fig. S9). In contrast, there were no genes that were top-scoring according to SPARK-X but missed by singleCellHaystack. Two examples of genes that were relatively higher-scoring for SPARK-X than for singleCellHaystack are *Zfhx3* (378th by singleCellHaystack vs 64th by SPARK-X) and *Cox4i1* (1123rd by singleCellHaystack vs 183rd by SPARK-X). Although both *Zfhx3* and *Cox4i1* show some degree of spatially differential expression, the tendency is weaker than those of *Plp1* and *Mal*.

In HDST datasets, singleCellHaystack and SPARK-X returned highly consistent results (Fig. 2F, Supplementary Fig. S10). For example, gene Gm42418 was the top-scoring SVG according to both methods in one mouse brain sample (Fig. 2F). Only 1 gene was found to have a discrepant result: Gm10925 was ranked 13th by singleCellHaystack (p-value 2.7 × 10^–39^) but only 105th by SPARK-X (p-value 0.013).

Finally, we compared both methods using three MERFISH datasets (Supplementary Fig. S11)^22^. This technology has the difficulty that the number of cells is small relative to other methods, making the identification of SVGs more challenging. In agreement with that, both singleCellHaystack and SPARK-X identified relatively lower numbers of differentially expressed genes.

In summary, top-scoring SVGs predicted by singleCellHaystack include clear cases that are missed by SPARK-X (Fig. 2B–F). The authors of SPARK-X noted that the assumptions made by SPARK-X are likely to be not optimal in detecting certain expression patterns^16^. Based on our experience, SPARK-X appears to work well on gradually changing patterns of expression, but suffers on patterns with abrupt differences between neighboring locations, exemplified by *Ttr* in Fig. 2C or *PLP1* in Fig. 2E.

So far, we have described applications to the multidimensional PC space of scRNA-seq data and 2D spatial coordinates of various spatial transcriptomics technologies. However, singleCellHaystack makes few assumptions about the underlying data distributions (distribution of read counts or UMIs, etc.). Because of the versatility of the density distribution and relative entropy approach on which singleCellHaystack is based, it is applicable to many other data types and input spaces. In the next sections we illustrate this general applicability using examples on scRNA-seq trajectory (1D) data, CITE-seq data, scATAC-seq data, a large collection of bulk RNA-seq data, and on gene set activity data.

### Predicting DEGs along trajectories

**Figure 3:**
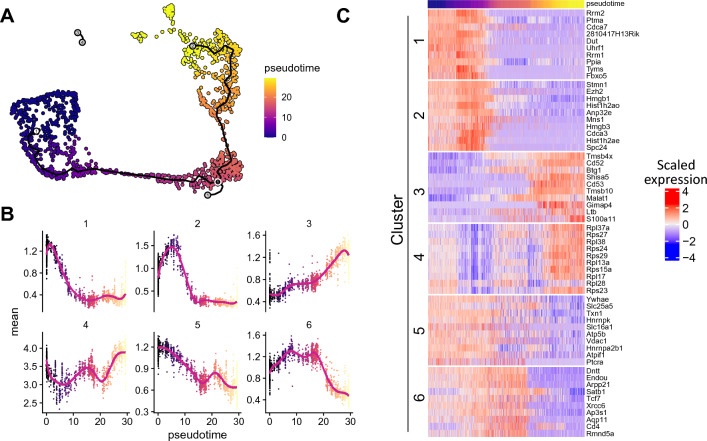
Application of singleCellHaystack to the prediction of DEGs along a trajectory. **(A)** UMAP plot of a Tabula Muris thymus dataset indicating the monocle3 trajectories and pseudotime. **(B)** Top scoring DEGs predicted by singleCellHaystack were clustered into modules. For each module the average expression of all genes at each pseudotime value is shown, indicating different patterns of expression changes along the trajectory. **(C)** For each module in panel **(B)**, the expression of the top 10 genes along the trajectory is shown in a heatmap.

We applied singleCellHaystack to 1D projections from trajectory pseudotime inference. To this end we used the thymus dataset from the Tabula Muris^28^, which contains data from developing thymocytes, progressing from a double negative (Cd4^−^Cd8^−^) through a double positive (Cd4^+^Cd8a^+^) stage into mature naive T cells characterized as single positive (i.e., either Cd4^+^ or Cd8a^+^). We processed the 10x Genomics Chromium data using the standard pipeline with Seurat^33^ and then used monocle3^34^ to order cells from the double negative cluster to the single positive clusters (Fig. 3A). We used this pseudotime ordering (a 1D space) as input for singleCellHaystack to identify DEGs with biased expression along this trajectory. In this case, singleCellHaystack generates grid points within this 1D input space, but otherwise proceeds in the same way as for datasets with 2 or more dimensions. To characterize the patterns and dynamics associated with the changes in expression we clustered the DEGs into 6 modules. The number of modules was chosen to be able to visualize a variety of expression patterns. Figure 3B shows the mean expression of the top-scoring genes in each module along the trajectory, whereas Fig. 3C shows the top 10 genes per module. These results indicate that singleCellHaystack can identify patterns of gene expression changes along trajectories.

### Applications to CITE-seq, scATAC-seq, and bulk RNA-seq data

The continuous version of singleCellHaystack is not restricted to single-cell transcriptomes but can be used with any numerical data. Here we demonstrate this by applying singleCellHaystack to CITE-seq, scATAC-seq and bulk RNA-seq data.

**Figure 4:**
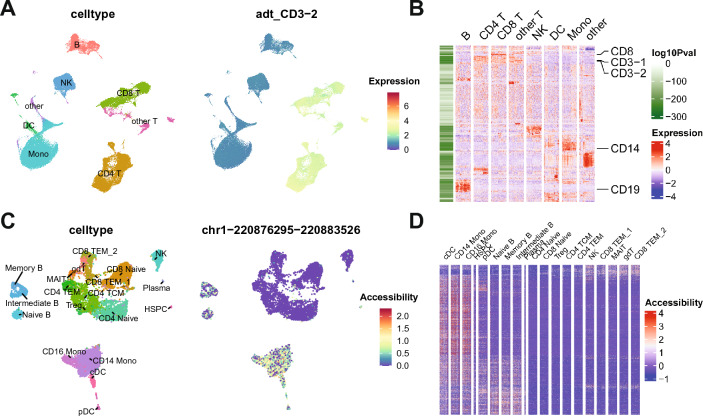
Example application to CITE-seq and scATAC-seq data. **(A)** A UMAP plot of the CITE-seq data with cell type annotations is shown (left) together with the top high-scoring protein predicted by singleCellHaystack (right). **(B)** A heatmap showing the expression levels of all proteins in a subset of cells, as well as the log p-values returned by singleCellHaystack (left bar). **(C)** A UMAP plot of the scATAC-seq data with cell types annotations is shown (left) together with the top high-scoring differentially accessible genomic region predicted by singleCellHaystack (right). **(D)** A heatmap showing the accessibility levels of the top 1000 high-scoring regions in a subset of cells.

To show singleCellHaystack applications to single-cell protein measurements we used a human peripheral blood mononuclear cell (PBMC) dataset containing the whole transcriptome, and the expression of more than 200 proteins^33^. We calculated PCA and UMAP coordinates, and cell clusters based on the expression of proteins and ran singleCellHaystack using 50 PCs and the protein counts. Figure 4A shows the UMAP plot with the clusters, and the top protein identified by singleCellHaystack. Figure 4B shows a heatmap with the expression level of all proteins in a subset of cells (maximum 100 cells per cluster). In the left side a bar indicates the log p-value returned by singleCellHaystack. Lower p-values are associated with stronger expression in one or more clusters compared to the rest. A subset of known cell markers is indicated to demonstrate that these proteins are indeed associated with the cell populations where they are expected to be expressed. Predictions made by singleCellHaystack were in general consistent with those made by cluster-based prediction approaches (Supplementary Fig. S12A,C) but differences in ranking could be seen for some proteins. For proteins with a clear difference in expression in one cluster, such as CD64 and CLEC12A, singleCellHaystack and cluster-based methods are in agreement (Supplementary Fig. S12C,D). However, proteins such as CD2 and CD3-1, which have a striking dichotomy in expression levels between several clusters of T-cells and NK cells as compared to several clusters representing B cells and myelocytes, were high-scoring according to singleCellHaystack, but less so according to the cluster-based approaches (Supplementary Fig. S12B,D). In contrast, cluster-based approaches tend to assign relatively low p-values to proteins with high or low expression in a single cluster, such as CD133-1 and CD133-2 in the case of the Wilcoxon rank sum test and CD303 and CD45-2 in the case of the t-test, even if the difference in expression levels is not visually obvious. Note that according to the t-test the differential expression of CD303 is judged to be of a similar statistical significance to that of CLEC12A. We made similar observations in a comparison between the binary approach and a cluster based approach applied to scRNA-seq data before^19^.

We also applied singleCellHaystack to a single-cell multiome (i.e., RNA and ATAC) dataset from human PBMCs downloaded from the 10x Genomics website (see “Materials and methods” section). We used the Signac package to process the RNA and ATAC counts. For ATAC we calculated a Latent Semantic Index (LSI) embedding and used it, with the peak counts, to identify DARs. Figure 4C shows the UMAP plot derived from the LSI embedding, together with the top genomic region identified by singleCellHaystack. Figure 4D shows a heatmap with the accessibility level of the 1000 high-scoring regions in a subset of cells (maximum 100 cells per cluster). This shows that the top DARs returned by singleCellHaystack have cluster-restricted activity patterns. Here too, the predictions made by singleCellHaystack were in general consistent with those made by cluster-based prediction approaches (Supplementary Fig. S13A–C). Generally, similar tendencies were seen as for the CITE-seq data (Supplementary Fig. S12). For example, the Wilcoxon test assigned low p-values to two regions which had a higher signal in the small cluster of pDCs (Supplementary Fig. S13B), and the t-test to regions with a lower signal in the myelocyte cluster (Supplementary Fig. S13D). In contrast, singleCellHaystack judged regions with a clearly distinct pattern between several T cells clusters and clusters representing other cells to be more significant (Supplementary Fig. S13B,D).

Another possible application is on large numbers of bulk RNA-seq samples. Here, as an example, we applied singleCellHaystack on a collection of 1958 RNA-seq samples obtained from various parts of the mouse brain (Supplementary Fig. S14A)^23^. singleCellHaystack successfully predicted DEGs with differential expression in subsets of the samples and generally showed consistency with other DEG prediction approaches such as the Wilcoxon rank sum test or the Student’s t-test (Supplementary Fig. S14C,D). However, similar to the CITE-seq and scATAC-seq examples above, because of their reliance on comparisons between clusters, both the Wilcoxon rank sum and the t-test tend to give a higher significance to genes with higher expression in a single cluster (Supplementary Fig. S14B,E,F).

### Predicting differentially active gene sets

**Figure 5:**
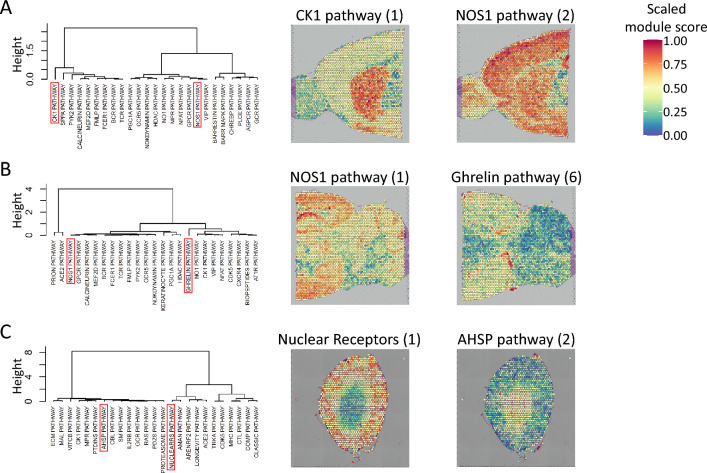
Application of singleCellHaystack to sets of genes. We applied singleCellHaystack on the module scores of sets of genes associated with 292 BioCarta pathways. For three spatial transcriptomics datasets, the top 25 high-scoring BioCarta pathways were clustered by similarity (left). Two example high-scoring pathways are indicated in red in the clustering result, and are visualized (right). Numbers in parentheses represent the rank of the p-value of the pathway (e.g. 1 indicates the most significant pathway). **(A)** In mouse anterior brain, **(B)** in mouse posterior brain, and **(C)** in mouse kidney.

Because singleCellHaystack makes few assumptions about the input data, it is not limited to applications to UMI or read count data but can be used with any quantitative data associated with the samples. As an illustration, we applied singleCellHaystack to module scores as computed by Seurat, which reflect the general activity of a set of genes. Here, as sets of genes, we used genes associated with 292 BioCarta pathways as defined in msigdbr^35^. For a number of spatial transcriptomics datasets, we calculated the module scores of each gene set in each Visium spot, and used singleCellHaystack to predict gene sets with highly non-random spatial distributions. Examples of high-scoring gene sets in mouse anterior and posterior brain and kidney tissue are shown in Fig. 5. We used hierarchical clustering to cluster the top 25 high-scoring gene sets by similarity (Fig. 5, left). For each dataset a variety of patterns was found, reflecting how different pathways are active in different parts of the tissues. Figure 5 (right) shows two example gene sets for each dataset, each showing high scores in distinct parts of the tissues.

## Discussion

In this manuscript we have presented a universally applicable tool for differential activity prediction in single-cell and spatial genomics data. This version of singleCellHaystack is an important improvement compared to existing DEG prediction tools and compared to the original implementation of singleCellHaystack. The original implementation required binary detection data as input (i.e. genes were treated as either detected or not in each cell), thus ignoring more subtle differences in expression between subsets of cells. At the same time, the use of a hard threshold can result in an overestimation of the significance of relatively modest differences in expression (see for example Fig. 1D, Supplementary Fig. S2). In this new version this issue has been addressed, and singleCellHaystack now uses continuous activity levels of features to detect DAFs. This enables singleCellHaystack to be used with any kind of continuous measurements, whether RNA or protein levels, chromatin accessibility or module scores. Furthermore, we showed that we can use singleCellHaystack with any kind of cell coordinates, whether they are physical spatial locations, PCA embeddings or pseudotime rankings.

Single-cell genomic datasets are rapidly increasing in numbers and in size, making it more challenging to perform exploratory analyses, including the identification of DAFs. Our new implementation of singleCellHaystack is significantly more efficient and faster than the original version, making it possible to analyze large datasets in a few minutes. For example, the Mouse Organogenesis Cell Atlas dataset with over 100 thousand cells took ~ 45 min to finish with the original version, whereas it takes around 5 min to finish with the new one (Supplementary Fig. S6C). Furthermore, our new Python implementation enables efficient identification of DAFs for atlas level datasets with millions of cells.

For spatial transcriptomics the fastest method available is SPARK-X. The short runtimes are accomplished by, among others, making several assumptions about the input data^16^. Possibly because of these assumptions, SPARK-X failed to identify several clear SVGs when compared to singleCellHaystack (see for example Fig. 2C). In contrast, we found no clear SVGs that were predicted by SPARK-X but not by singleCellHaystack. The better sensitivity of singleCellHaystack makes it the best alternative, despite relatively longer runtimes.

Different methods for the identification of DAFs are being used depending on the technology. For example, Wilcoxon rank sum tests and t-tests are used to identify DEG between groups of cells. Moran’s I and other methods are used to identify SVGs in spatial transcriptomics and trajectory analyses. In this paper we show that singleCellHaystack is not restricted by the technology or by the particularities of features that were measured, nor by the type of coordinates that were used as input. This makes singleCellHaystack a universal tool for the identification of DAFs.

Despite its advantages, singleCellHaystack has a few weak points. One is that comparisons between multiple conditions (e.g., wild-type and knockout) cannot be easily conducted. We hope to expand singleCellHaystack to include methods for such comparisons in the future. Secondly, for better or worse, current scRNA-seq data analyses are often cluster-oriented. Methods that are based on making comparisons between clusters (such as the Wilcoxon rank sum test and the t-test) tend to have a bias towards features with activity patterns that fit well with one of a few clusters, but are likely to miss features whose differential activity is spread out over several clusters (Supplementary Figs. S12–S14). We found similar tendencies in a previous comparison^19^. In contrast, singleCellHaystack is able to detect more complex patterns of activity. However, the clustering of cells is a convenient tool for summarizing complex datasets and for performing additional downstream tests. Compared to cluster-based DEG prediction approaches, it is not as straightforward to incorporate the results of singleCellHaystack into a cluster-oriented scRNA-seq analysis. However, other fields of genomics, such as spatial transcriptomics, are less focused on clustering, and not all scRNA-seq datasets can be easily summarized by clustering. In the future, we hope to develop approaches to integrate singleCellHaystack results with the information from clusters.

In conclusion, singleCellHaystack is a fast, data type agnostic DAF prediction method. Because it doesn’t rely on comparisons between clusters, it has the sensitivity needed to detect complex patterns of activity. The density distribution and relative entropy approach provides the versatility to handle a wide range of applications. We believe that singleCellHaystack will play a valuable role in future exploratory analysis of single-cell genomics datasets.

## Materials and methods

### singleCellHaystack methodology

For a detailed description of the original singleCellHaystack implementation (version 0.3.2) we refer to Vandenbon and Diez^19^. In brief, singleCellHaystack uses the distribution of cells inside an input space to predict DAFs. First, it infers a reference distribution $Q$ of all cells in the space by estimating the local density of cells surrounding a set of grid points in the space. In a next step, the original singleCellHaystack estimated the distribution of the cells in which a gene $G$ is detected (distribution $P(G=T)$) and not detected (distribution $P(G=F)$). The Kullback–Leibler divergence (${D}_{KL}$) was used to compare $P(G=T)$ and $P(G=F)$ to the reference distribution $Q$. The statistical significance of each gene’s ${D}_{KL}$ was evaluated using random sampling.

The updated version of singleCellHaystack (version 1.0.0) includes several improvements. The main improvement is that singleCellHaystack no longer treats expression in a binary way (i.e. detected or not detected), but uses continuous values (see Steps 3–4 below). Secondly, we updated the modeling of ${D}_{KL}$ values using splines. In the new implementation, we use cross-validation to select a suitable flexibility of the splines (see Step 5). The new implementation also accepts input data as sparse matrices, and a Python implementation has been made available. Below follows a more detailed description of the singleCellHaystack version 1.0.0 methodology.

#### Step 1: setting parameters

The main inputs to singleCellHaystack are the coordinates of samples inside an input space, and the observations in each sample. Here, samples include single cells, spots, pucks or even bulk samples, depending on the platform used. The input space could be the 2D or 3D space in a tissue or a latent space after typical dimensionality reduction (e.g. first principal components of a scRNA-seq dataset), or the 1D coordinates of samples along a trajectory (pseudotime). Coordinates of samples in $d$-dimensional space will be denoted as ${s\in R}^{d}$*.* By default, the coordinates in each dimension are rescaled to mean 0 and standard deviation 1. Observations might be gene expression, chromatin accessibility, gene set module scores, etc. and will be denoted as $y$.

Steps 2–4 involve estimating the distribution of samples inside the $d$-dimensional input space by estimating the local density of samples around a set of grid points using a Gaussian kernel. Ideally, grid points should cover the entire subspace occupied by the samples, and they should be spread out uniformly over this subspace, without gaps in their coverage. This topic is related to space-filling design algorithms, such as Latin hypercube design and minimax distance design^36^. Such approaches, however, are not necessarily suitable for high-dimensional spaces nor for spaces that are partly empty. In our methods, by default, the coordinates of grid points are instead decided by running k-means clustering on the sample coordinates $s$ and then using the resulting centroids as grid points. This approach tends to result in grid points being roughly uniformly spread over the subspace of the input space where samples are located. Note that the goal of this step is not to obtain clusters of cells, but merely to obtain suitable grid points. Furthermore, note that the grid point coordinates are based on the coordinates of samples in the input space, and not on the activity levels of the features. By default, singleCellHaystack uses $g$ = 100 grid points (option grid.points). This number can be reduced (e.g. when the number of samples is low) or increased (for highly heterogeneous datasets or when the number of samples is high) as needed. We have shown before that results are stable w.r.t. the number of grid points^19^. Alternatively, the user can specify the coordinates of the grid points to use (option grid.coord), or use seeding, as used in k-means++ clustering, as described before (grid.method = “seeding”)^19^.

The bandwidth $h$ of the Gaussian kernel is set as before^19^. For each sample, the Euclidean distance to the closest grid point is calculated, and $h$ is defined as the median of those distances. Normalized distances between samples and grid points are subsequently defined as the Euclidean distances divided by the bandwidth $h$. The density contribution ${d}_{i,j}$ of each sample $i$ to each grid point $j$ is calculated as:

$${d}_{i,j}={e}^{\left(- \frac{{{dist}_{i,j}}^{2}}{2}\right)},$$

where ${dist}_{i,j}$ is the normalized distance between sample $i$ and grid point $j$.

#### Step 2: estimating reference distribution Q

The reference distribution $Q=({Q}_{1},\dots ,{Q}_{g})$ of all $n$ samples in a dataset is estimated as described before^19^. In brief, the density of cells around grid point $j$ is calculated as the sum of all ${d}_{i,j}$ values:

$${Q}_{j}=\sum_{i=1}^{n}{d}_{i,j}.$$

After this, $Q$ is normalized to sum to unity.

#### Step 3: estimating ${P}_{f}$ distributions

Whereas the original version of singleCellHaystack treated observations in a binary way (a gene is either detected or not detected in each cell), this updated version of singleCellHaystack addresses this weak point and treats observations in a continuous manner. To do so, the distribution ${P}_{f}=({P}_{f,1},\dots ,{P}_{f,g})$ of feature $f$ in the input space is calculated as follows:

$${P}_{f,j}=\sum_{i=1}^{n}{d}_{i,j}{y}_{f,i},$$

where ${d}_{i,j}$ is the density contribution of sample $i$ to grid point $j$, and ${y}_{f,i}$ is the activity of feature $f$ in sample $i$. ${P}_{f,j}$ is therefore the sum of density contributions of samples to grid point $j$ weighted by the activity of $f$. Subsequently, ${P}_{f}$ is normalized to sum to unity.

#### Step 4: estimating the Kullback–Leibler divergence of feature $f$, ${D}_{KL}(f)$

The divergence of feature $f$, ${D}_{KL}(f)$, is calculated as follows:

$${D}_{KL}(f)=\sum_{j=1}^{g}{P}_{f,j}\mathrm{log}\left(\frac{{P}_{f,j}}{{Q}_{j}}\right).$$

This approach is simpler than the original version, because no distinction needs to be made between samples in which a feature was detected or not detected^19^. If the activity of feature $f$ does not show a biased distribution, and approximately follows the reference distribution $Q$, then ${D}_{KL}(f)$ is close to 0. As the discrepancy with the reference distribution $Q$ increases, the value of ${D}_{KL}(f)$ also increases.

#### Step 5: estimating the significance of ${D}_{KL}(f)$

In a final step, singleCellHaystack evaluates the statistical significance of ${D}_{KL}(f)$ values by comparing them to randomized data. In principle, it would be possible to generate many randomly shuffled sets of activity values of each feature $f$, and use these to estimate a null distribution of randomized ${D}_{KL,random}(f)$ values. However, doing this for each feature would be prohibitively time-consuming. Instead, singleCellHaystack uses the following approach.

First, singleCellHaystack calculates the coefficient of variation (CV = standard deviation/mean) of each feature $f$. Features are ordered by CV, and a subset of features (100 by default) that are spread evenly over the range of CV values is selected. These features are used for making randomly permutated datasets (100 by default for each selected feature) based on which ${D}_{KL,random}(f)$ values are calculated.

For each randomized feature $$f$$, the $${log(D}_{KL,random}(f))$$ values follow an approximately normal distribution. This allows us to use their mean and standard deviation to estimate p-values of actually observed $${D}_{KL}(f)$$ values. Moreover, we can use CV values as predictor of the mean and standard deviations of $${log(D}_{KL,random}(f))$$ values. In singleCellHaystack, by default we model the mean and standard deviation of the $${log(D}_{KL,random}(f))$$ values in function of $$log($$CV$$)$$ values using natural cubic splines. Splines are trained using function ns in the splines R package. A suitable degree of freedom (between 1 and 10) is decided using tenfold cross-validation.

Alternatively, B-splines can be used, using function bs. In this case, a suitable degree (between 1 and 5) and degree of freedom (between 1 and 10) is decided in the same way.

Using the splines, the expected mean and standard deviation of ${log(D}_{KL,random}(f))$ are predicted for every feature, in function of its CV, and based on that the corresponding p-value of ${D}_{KL}(f)$ using the pnorm function in R.

### Tabula Muris and Mouse Cell Atlas scRNA-seq datasets

We obtained the Tabula Muris data from https://figshare.com/articles/dataset/Robject_files_for_tissues_processed_by_Seurat/5821263 (version 3)^28^. For the Mouse Cell Atlas data, we downloaded file MCA_BatchRemove_dge.zip from https://figshare.com/articles/MCA_DGE_Data/5435866^29^. This data has been treated to reduce batch effects. Data from both Tabula Muris (32 datasets) and Mouse Cell Atlas (87 datasets) were processed using the Seurat R package (version 4.0.0)^33^. We used the same pipeline for the processing and normalization of all datasets: genes detected in less than 3 cells and cells with fewer than 100 detected genes were removed. After this initial filtering, cells with extreme UMI counts (bottom 1 percentile and top 1 percentile), or extreme numbers of detected genes (bottom 1 percentile and top 1 percentile), or with a high fraction of mitochondrial reads (> 10%) were removed. The data for the remaining cells of each dataset was normalized (NormalizeData, default settings), scaled (ScaleData, regressing out the UMI count and mitochondrial fraction), and highly variable genes (HVGs) were detected (FindVariableFeatures, default settings). The HVGs were used for principal component analysis (PCA), and the 20 first principal components (PCs) were used for further dimensionality reduction (t-SNE and UMAP) and for clustering of cells (FindNeighbors and FindClusters, using 20 PCs and otherwise default settings). Both the binary (version 0.3.2) and updated (version 1.0.0) singleCellHaystack were applied on the first 20 PCs of each dataset. For the binary version, in each dataset, for each gene, the median expression level of each gene was used as a threshold to define detection. The detection data was used as input. For the updated version, the continuous expression levels were used as input.

We conducted a comprehensive comparison between genes that are high-scoring according to either the continuous or the binary version of singleCellHaystack (but not both) as follows. From the prediction results of the 119 Tabula Muris and Mouse Cell Atlas datasets, we picked up genes that were among the top 250 high-scoring genes according to one approach, but were ranked at least 1000 ranks lower by the other approach. We calculated the mean expression and coefficient of variation (= standard deviation/mean) of these genes in the dataset in which they were predicted to be DEGs (Supplementary Fig. S1).

For the analysis of the effect of sample sizes, we used the 29 Tabula Muris and Mouse Cell Atlas datasets that contained more than 5000 cells. For each dataset, we randomly selected 100, 200, 300, 400, 500, 1000 and 2000 cells, and conducted normalization and dimensionality reduction using PCA as described above. We predicted DEGs with singleCellHaystack using the first 20 PCs as input space. For comparison, we used the same 20 PCs to cluster cells, and predicted DEGs using Seurat’s FindAllMarkers function (using the default Wilcoxon rank sum test). For each down-sampled dataset, we collected the p-values of all genes as estimated by singleCellHaystack and FindAllMarkers (when a gene had multiple p-values we collected the smallest one). Finally, we compared results of the down-sampled datasets to the results of the full datasets using the Spearman’s rank correlation of the p-values as a measure of the robustness of the results (Supplementary Fig. S3).

For the analysis of the sensitivity of the continuous and binary versions of singleCellHaystack w.r.t. the input space, we applied both version to the Tabula Muris lung dataset, using as input space the first 5, 20, or 50 PCs.

### Large scRNA-seq dataset

We downloaded scRNA-seq expression data for 35,686 transcripts in 4,062,980 cells of fetal tissues^30^. For the analysis we used our Python implementation of singleCellHaystack (https://github.com/ddiez/singleCellHaystack-py) using PCA coordinates with 50 components. To check scalability with dataset size we compared the runtimes obtained with the R and Python implementations, using datasets containing 1000, 10,000, 50,000, 100,000, 500,000 and 1 million randomly selected cells from this dataset (Supplementary Fig. S6C).

### Trajectory analysis

We applied singleCellHaystack to pseudotime projection on the Tabula Muris thymus data^28^. In this dataset the development of T cells can be followed from double negative (CD4^−^CD8^−^), through double positive (CD4^+^CD8^+^) and into mature, single positive (CD4^+^CD8^−^ and CD4^−^CD8^+^) T cells. To identified the differentiation trajectory we used monocle3^34^. Briefly, the data was first processed using the standard Seurat pipeline (see above), except that 30 PCs were used to calculate UMAP coordinates. We converted the Seurat object into a cell_data_set object with the SeuratWrappers package (https://github.com/satijalab/seurat-wrappers). Then monocle3 was used to calculate clusters and partitions using the UMAP coordinates with the function cluster_cells. Next, the principal graph is learned using the learn_graph function, and cell were ordered selecting as root the node in the graph starting in the cluster of double negative cells. We used singleCellHaystack using the pseudotime coordinates. We selected the top 1,000 predicted DEGs and clustered them into modules using kmeans, using k = 6.

### CITE-seq dataset

Single-cell data from human peripheral blood mononuclear cells (PBMC) data was downloaded from https://atlas.fredhutch.org/nygc/multimodal-pbmc/. This dataset contains information about the expression of 228 immune marker proteins on over 200 k cells. As input to singleCellHaystack we used the protein based PCA coordinates (50 PCs), and the normalized expression levels included in the downloaded data, which was processed as described here^33^. To compare with singleCellHaystack, differentially expressed proteins were also predicted using the default Wilcoxon rank sum and the Student’s t-test implemented in Seurat’s FindAllMarkers function, with default parameters except max.cells.per.ident = 500, and the original cell type annotations (celltype.l2) as clusters.

### scATAC-seq dataset

Single-cell multiome (RNA + ATAC) data from human PBMC was downloaded from the 10x Genomics web site (https://support.10xgenomics.com/single-cell-multiome-atac-gex/datasets/1.0.0/pbmc_granulocyte_sorted_10k). The raw data (fragments and peak information from cellranger) were processed with Signac^37^, following the workflow described here: https://satijalab.org/seurat/articles/weighted_nearest_neighbor_analysis.html#wnn-analysis-of-10x-multiome-rna-atac-1. Briefly, the expression and chromatin accessibility peak information data were loaded into a Seurat object. For the peaks, the information about genomic ranges was obtained using the function GetGRangesFromEnsDb with the Bioconductor package EnsDb.Hsapiens.v86 (http://bioconductor.org/packages/EnsDb.Hsapiens.v86/). Cells were filtered to have less than 20% of mitochondrial counts, RNA counts between 1000 and 25,000 and ATAC counts between 5 × 10^3^ and 7 × 10^7^. For the RNA data the SCTransform pipeline was used, and UMAP coordinates were calculated using 50 PCs. For the ATAC counts, first term-frequency inverse-document-frequency was calculated with RunTFIDF. Top features were selected with FindTopFeatures and min.cutoff = “q0”. Then, a Latent Semantic Index (LSI) embedding was calculated with RunSVD. ATAC based UMAP was constructed from dimensions 2 to 50 from LSI. singleCellHaystack was run using LSI embedding and ATAC peak counts. To compare with singleCellHaystack, differentially accessible regions (DARs) were also predicted using the default Wilcoxon rank sum and the Student’s t-test implemented in Seurat’s FindAllMarkers function, with default parameters except max.cells.per.ident = 500, and the original cell type annotations as clusters.

### Spatial transcriptomics datasets

We obtained and processed data for the following four platforms (Table 1).

#### Visium platform data

Data for mouse kidney and brain were obtained through the SeuratData R package^38^. We filtered out mitochondrial genes and genes with non-zero counts in less than 10 spots. Data was normalized using the Seurat R package (function NormalizeData, with default parameters).

#### Slide-seqV2 data

We obtained the data from the Broad Institute Single Cell Portal (accession number SCP815)^20^. We filtered out mitochondrial genes and genes with non-zero counts in less than 10 spots, as well as pucks with less than 100 reads in total. Data was normalized using the Seurat R package (function NormalizeData, with default parameters).

#### HDST data

We obtained the data from the Broad Institute Single Cell Portal (accession number SCP420)^21^. We filtered out mitochondrial genes and genes with non-zero counts in less than 10 spots. Data was normalized using the Seurat R package (function NormalizeData, with default parameters).

#### MERFISH data

RNA counts and cell position information was obtained from Xia et al. (Datasets S12 and S15)^22^. We filtered out genes with non-zero counts in less than 10 spots, as well as cells with less than 6000 detected genes. Data was normalized using the Seurat R package (function NormalizeData, with default parameters).

We applied the following methods on each of the spatial datasets: the updated version of singleCellHaystack, SPARK, SPARK-X, Seurat’s FindSpatiallyVariableFeatures function using Moran’s I and mark variogram approaches, MERINGUE, and Giotto’s binSpect using the kmeans and the rank approaches^12,15–17,19,31,32^. Because several methods have long runtimes or returned errors on large datasets (Fig. 2A), we limited the analysis to the top 1000 HVGs (detected using function FindVariableFeatures). In addition, we applied singleCellHaystack and SPARK-X on all genes in the datasets. To each method, we gave as input the same 2D spatial coordinates of the samples, along with the expression data (counts or processed data as needed). Each method was run with default parameter settings on the 1000 HVGs (without other pre-filtering steps), and the number of cores used was set to 1, to make the comparison of runtimes fair. Method-specific normalization steps were not included in the runtimes. SPARK was applied using CreateSPARKObject (option percentage and min_total_reads set to 0), spark.vc (with covariates set to NULL and library sizes set to the total number of counts per sample) and spark.test. SPARK-X was run with function sparkx (option set to “mixture”). MERINGUE was run using functions normalizeCounts, getSpatialNeighbors, and getSpatialPatterns. Seurat’s FindSpatiallyVariableFeatures was run with assay set to “Spatial”. Giotto was applied using functions createGiottoObject, normalizeGiotto, createSpatialNetwork, and binSpect (with bin_method set to “kmeans” or “rank” and got_av_expr and get_high_expr set to FALSE).

### Large collection of bulk RNA-seq data

We downloaded a large collection of RNA-seq samples covering 76 mouse cell types and tissues^23^. This data has been normalized using Upper Quartile normalization and treated for batch effects using ComBat^39,40^. From this dataset, we selected samples obtained from brain, prefrontal cortex, hippocampus, cortex, frontal cortex, olfactory bulb, cerebellum, forebrain, neocortex, and cerebral cortex. We treated the data using Seurat, including scaling, finding 1000 HVGs, and PCA. We filtered out genes with generally low expression (mean expression in the bottom 25%), and applied singleCellHaystack on the resulting 1958 samples and 18,260 genes, using as input space the first 5 PCs, using 25 grid points and otherwise default parameters. For visualization purposes, we applied UMAP on the first 5 PCs. For comparison, DEGs were also predicted using the default Wilcoxon rank sum and the Student’s t-test implemented in Seurat’s FindAllMarkers function, after clustering samples using default parameters.

### Module scores of gene sets

We used the R package msigdbr (version 7.5.1) to retrieve sets of mouse genes associated with BIOCARTA pathways^35^. For 292 pathways which had at least 10 associated genes, we collected their genes, and used the Seurat function AddModuleScore to calculate module scores in the spots of the Visium datasets. These module scores reflect the general activity of the genes in each pathway. Subsequently, we ran singleCellHaystack on each Visium datasets, using as input the coordinates of spots and the module scores (rescaled to be in the range 0 to 1) of all pathways. High-scoring pathways thus reflect pathways with spatial differences in activity within the tissue. The top 25 high-scoring pathways in each dataset were clustered by similarity using function hclust_haystack of the singleCellHaystack package.

## Supplementary Information

Supplementary Information.

## Data Availability

Single-cell RNA-seq datasets analyzed in this study are available from https://figshare.com/articles/dataset/Robject_files_for_tissues_processed_by_Seurat/5821263 (Tabula Muris), https://figshare.com/articles/MCA_DGE_Data/5435866 (Mouse Cell Atlas), https://atlas.fredhutch.org/nygc/multimodal-pbmc/ (PBMC CITE-seq), https://support.10xgenomics.com/single-cell-multiome-atac-gex/datasets/1.0.0/pbmc_granulocyte_sorted_10k (PBMC scRNA-seq and scATAC-seq). Visium data was obtained through the SeuratData package. Slide-seqV2 and HDST data from the Broad Institute Single Cell Portal, accession numbers SCP815 and SCP420. MERFISH data from the Supplementary Data in Xia et al. Bulk RNA-seq data was downloaded from https://figshare.com/articles/dataset/Mouse_data/14178425/1.

## Code availability

singleCellHaystack is implemented as an R package and is available from CRAN (https://CRAN.R-project.org/package=singleCellHaystack) and GitHub (https://github.com/alexisvdb/singleCellHaystack), and as a Python package available from PyPI (https://pypi.org/project/singleCellHaystack) and GitHub (https://github.com/ddiez/singleCellHaystack-py).
