# Supplementary material for: A universal tool for predicting differentially active features in single-cell and spatial genomics data
Source: Sci Rep. 2023 Jul 22;13:11830. doi: 10.1038/s41598-023-38965-2 (PMC10363154; doi:10.1038/s41598-023-38965-2)
Supplement: Supplementary file 1 — Supplementary Information. [file 41598_2023_38965_MOESM1_ESM.pdf]

Supplementary Information for

A universal tool for predicting differentially active features in single-cell and spatial genomics data

Alexis Vandenbon[1,2,$,*] and Diego Diez[3,4,$]

[1] Institute for Life and Medical Sciences, Kyoto University, 53 Shougoin Kawahara-cho, Sakyo-ku, Kyoto 606-8507, Japan.
[2] Institute for Liberal Arts and Sciences, Kyoto University, Yoshidanihonmatsu-cho, Sakyo-ku, Kyoto 606-8501, Japan.
[3] Immunology Frontier Research Center, Osaka University, 3-1, Yamada-oka, Suita, Osaka 565-0871, Japan.
[4] Open and Transdisciplinary Research Institute (OTRI), Osaka University, 1-1, Yamada-oka, Suita, Osaka 565-0871, Japan.

*Corresponding author. Email: alexisvdb@infront.kyoto-u.ac.jp

[$] These authors contributed equally to this work.

Table of Contents

A universal tool for predicting differentially active features in single-cell and spatial genomics data 1
SUPPLEMENTARY RESULTS 2
Comprehensive comparison of the binary and continuous versions of singleCellHaystack 2
Dependency of singleCellHaystack on sample sizes 2
SUPPLEMENTARY FIGURES 3
SUPPLEMENTARY REFERENCES 17

SUPPLEMENTARY RESULTS

Comprehensive comparison of the binary and continuous versions of singleCellHaystack

We applied the binary version of our method (singleCellHaystack version 0.3.2) and the updated continuous approach (singleCellHaystack version 1.0.0) to 119 scRNA-seq datasets of Tabula Muris and Mouse Cell Atlas [1,2]. As described in the main paper, both methods returned generally consistent results (section “Introduction to the singleCellHaystack method”, Fig. 1B). However, a number of genes were regarded as high-scoring differentially expressed genes (DEGs) by one approach but not by the other. Here, in each of the 119 datasets, we picked up genes that were among the top 250 high-scoring genes according to one approach, but were ranked at least 1000 ranks lower by the other approach. To gain insights into the common properties of these DEGs, we calculated their mean expression and coefficient of variation (= standard deviation / mean) in the dataset in which they were predicted to be DEGs. Results are summarized in Supplementary Figure S1. In general, genes that were high-ranking according to the binary version (but not the continuous version) had higher mean expression but little variation of expression. In contrast, genes that were high-ranking according to the continuous version (but not the binary version) had lower mean expression but more variation of expression. Note that the genes shown in Fig. 1C-D and Supplementary Figure S2 also follow this pattern.

Dependency of singleCellHaystack on sample sizes

To explore the sensitivity of singleCellHaystack with regard to sample sizes, we conducted the following analysis. We selected the 29 scRNA-seq datasets from the Mouse Cell Atlas and Tabula Muris projects that contain >5,000 cells. We next randomly down-sampled each dataset to smaller sizes (100, 200, 300, 400, 500, 1000, and 2000 cells). For each down-sampled dataset, we ran the standard processing workflow in Seurat (i.e., predict highly variable features and run PCA), and predicted DEGs using singleCellHaystack using the first 20 PCs. Finally, we compared results of the down-sampled datasets to the results of the full datasets using the Spearman’s rank correlation of the p-values as a measure of the robustness of the results. For comparison, we did a similar comparison for the Wilcoxon rank sum test implemented in Seurat’s FindAllMarkers function. For this, we used the same 20 PCs to cluster cells, and predicted DEGs using FindAllMarkers (see Methods for more details). Results are summarized in Supplementary Fig. S4.

As expected, for both methods the consistency decreased with smaller sample sizes. However, in general, singleCellHaystack returned more robust results (i.e., the Spearman rank correlations were higher) than Seurat’s FindAllMarkers approach.

SUPPLEMENTARY FIGURES

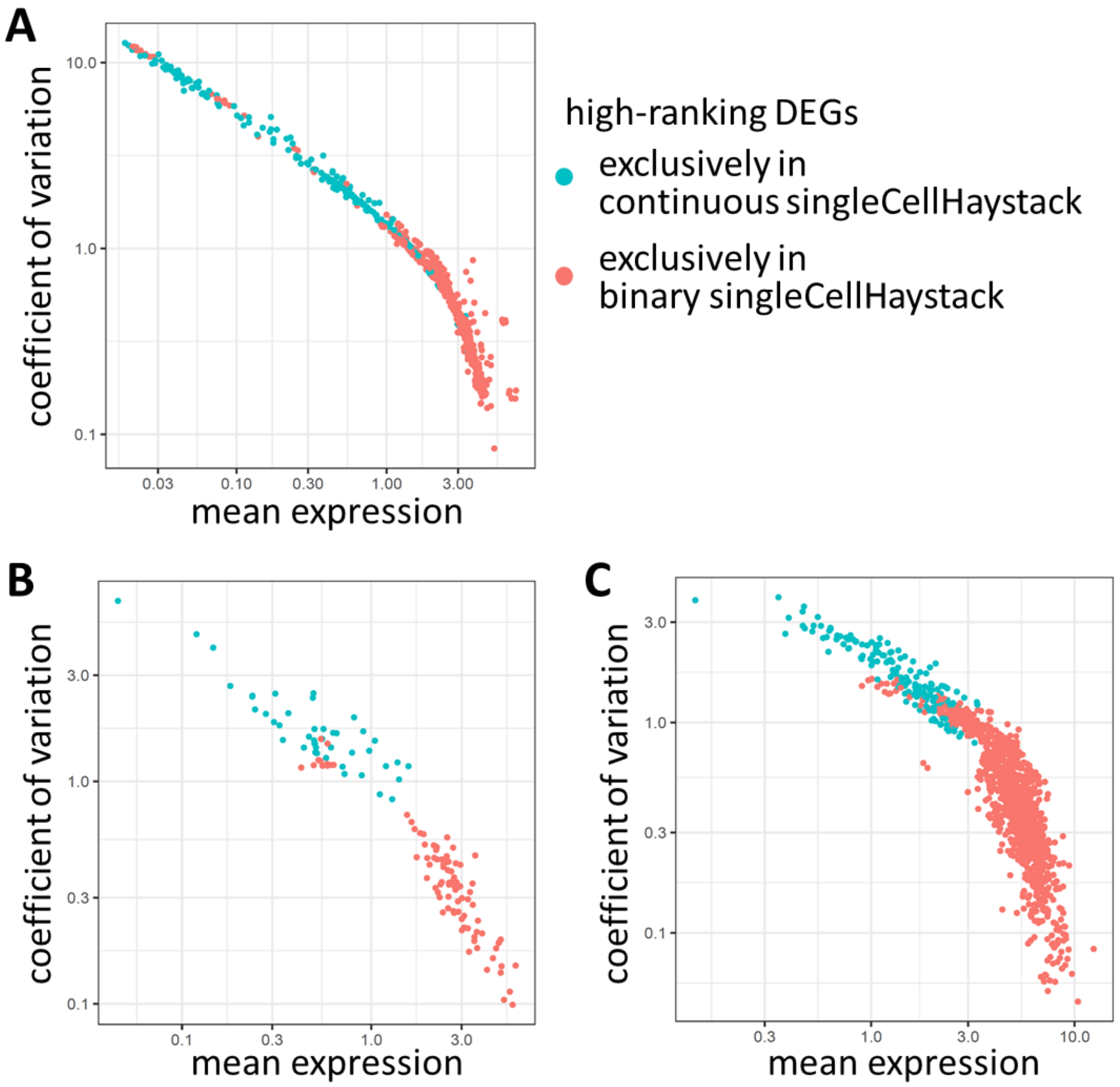

Supplementary Figure S1: Mean expression and coefficient of variation (= standard deviation / mean) of DEGs which were high-ranking exclusively according to the continuous (blue) or the binary (red) versions of singleCellHaystack. Each dot represents a DEG predicted in one of the datasets. Plots are shown for datasets generated by the Mouse Cell Atlas project **(A)**, and Tabula Muris project using microfluidic droplets **(B)** and FACS-sorted cells **(C)**.

(next page) **Supplementary Figure S2:** Additional examples of differences between the original binary method and the new continuous method of singleCellHaystack. For 6 Tabula Muris tissues, examples are shown of genes that are high-scoring according to the new continuous singleCellHaystack but not according to the original binary approach (left side), and vice versa (right side). For each gene, the gene symbol and the ranks according to the continuous (“cont”) and binary (“bin”) approaches are shown, as well as tSNE plots with the normalized expression levels used by the continuous version (top) and binarized detection levels as used by the binary approach (bottom).

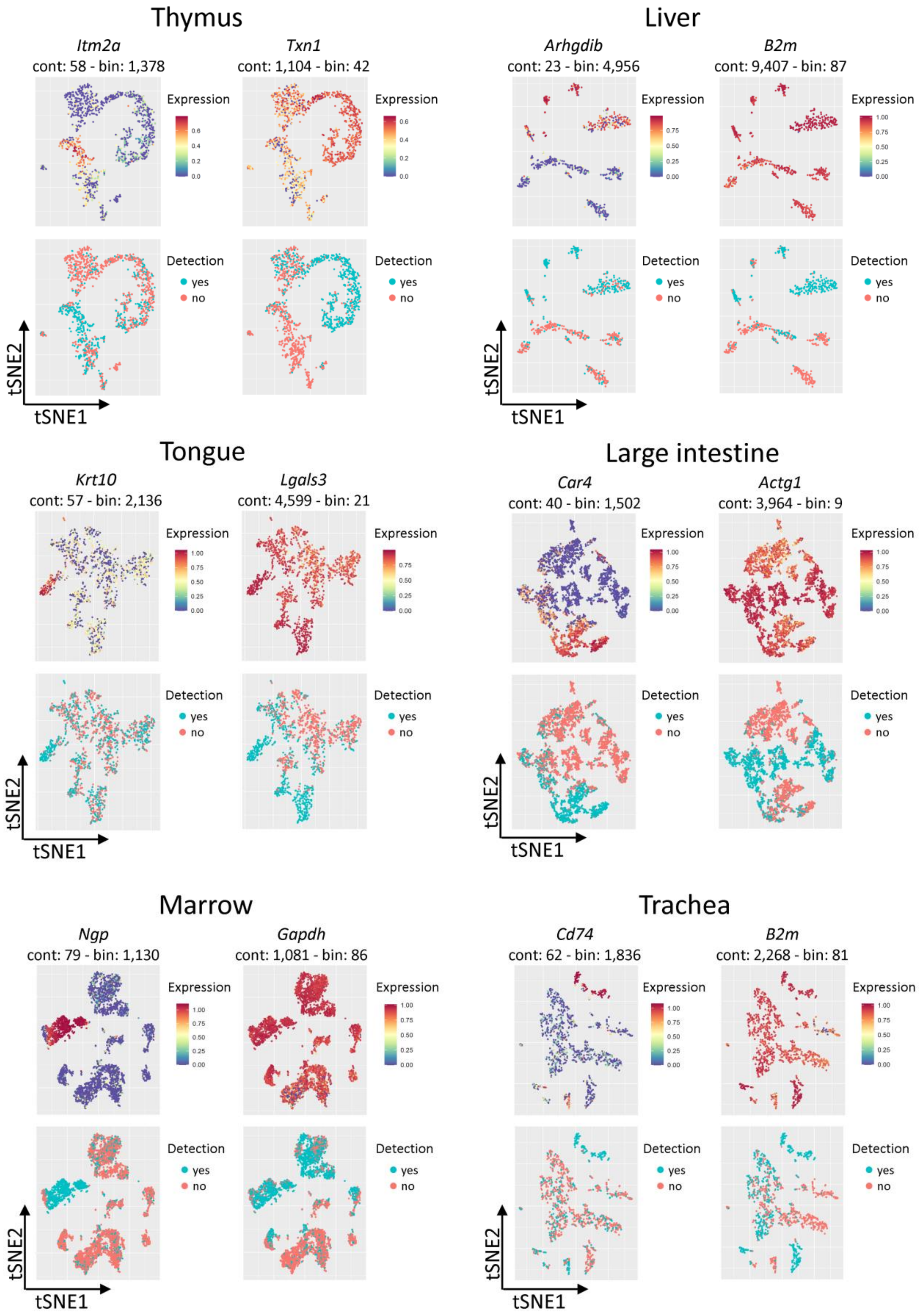
Thymus
Itm2a
cont: 58 - bin: 1,378
Txn1
cont: 1,104 - bin: 42
Liver
Arhgdib
cont: 23 - bin: 4,956
B2m
cont: 9,407 - bin: 87
Tongue
Krt10
cont: 57 - bin: 2,136
Lgals3
cont: 4,599 - bin: 21
Large intestine
Car4
cont: 40 - bin: 1,502
Actg1
cont: 3,964 - bin: 9
Marrow
Ngp
cont: 79 - bin: 1,130
Gapdh
cont: 1,081 - bin: 86
Trachea
Cd74
cont: 62 - bin: 1,836
B2m
cont: 2,268 - bin: 81
Expression
Detection
yes
no
tSNE2
tSNE1

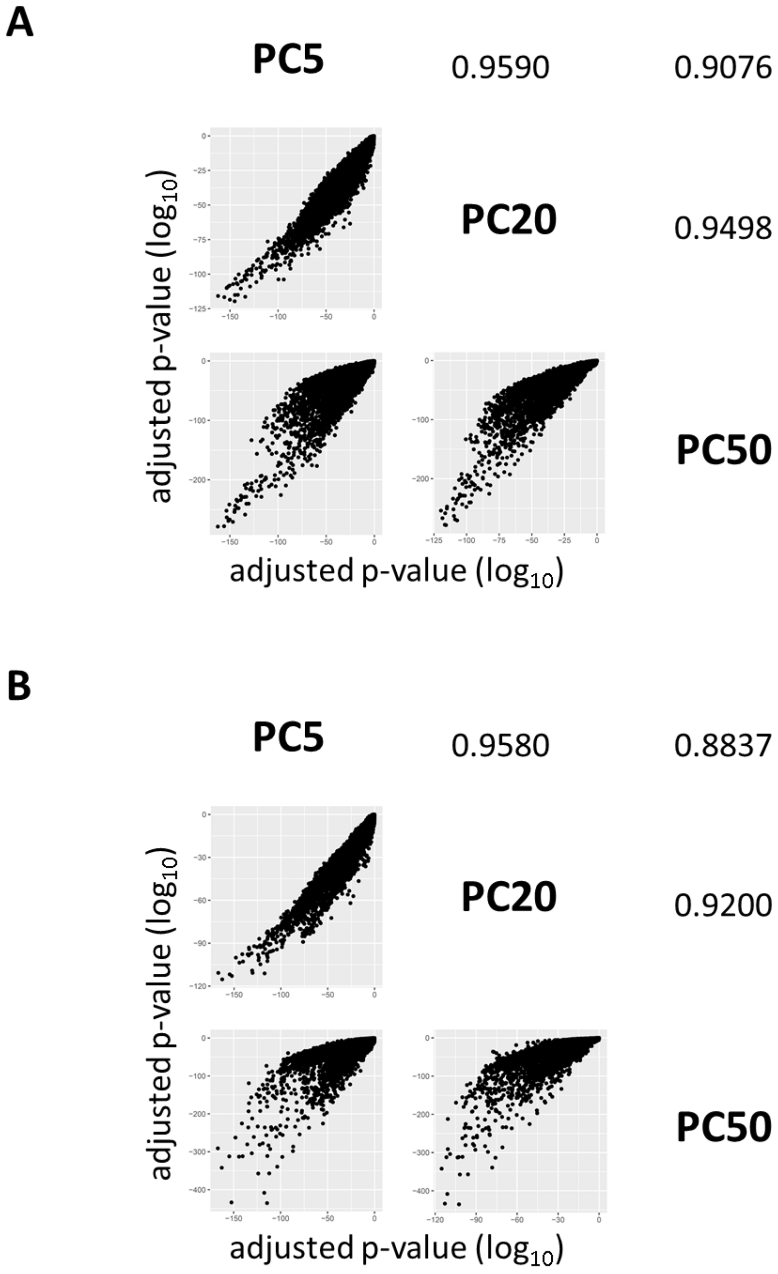

Supplementary Figure S3: Comparison of the robustness of the binary and continuous versions of singleCellHaystack w.r.t. input space. Both versions were applied to the Tabula Muris lung tissue dataset using the first 5, 20, or 50 PCs. Shown are scatterplots of the adjusted p-values ($\log_{10}$) for pairs of input spaces along with the corresponding Spearman's rank correlation, for the binary **(A)** and the continuous **(B)** versions of singleCellHaystack.

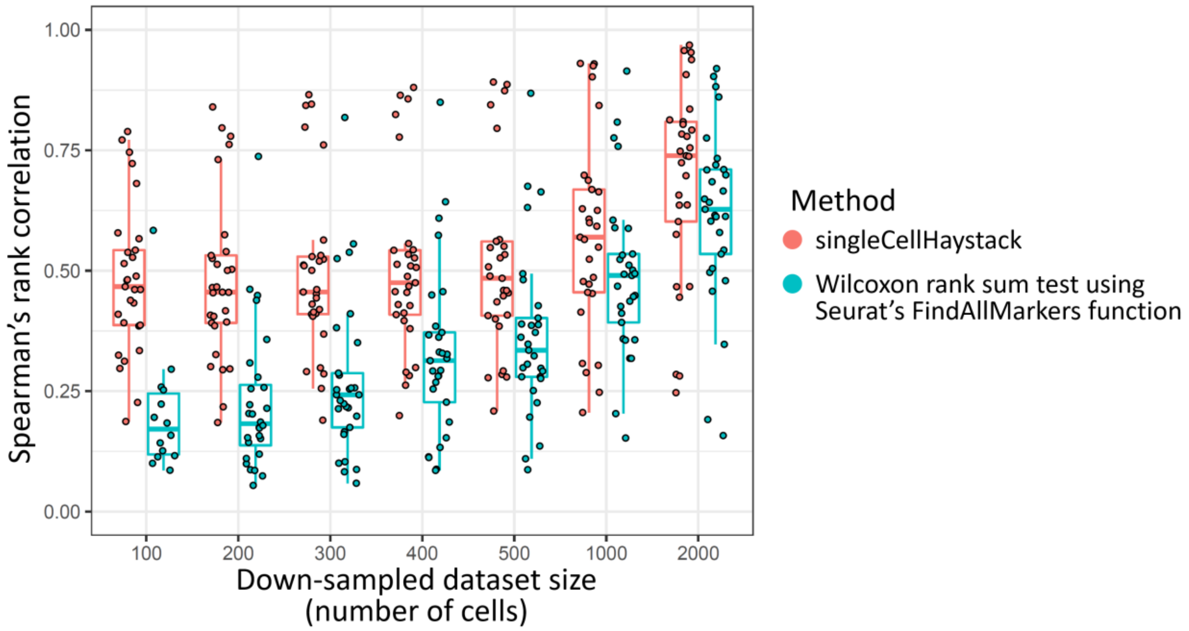

Supplementary Figure S4: Analysis of the robustness of singleCellHaystack w.r.t. sample size. We predicted DEGs in 29 scRNA-seq datasets that contain >5,000 cells, as well as on randomly down-sampled datasets of different sizes (X-axis), using singleCellHaystack and the Wilcoxon rank sum test implemented in Seurat's FindAllMarkers function. Boxplots show the consistency of DEG prediction results between the full datasets and the down-sampled datasets, for both methods. The consistency is estimated using the Spearman's rank correlation (Y axis) between log p-values. Note that for down-sampled datasets of 100 cells and 200 cells, Seurat predicted only a single cluster in 15 and 1 of the 29 datasets, respectively. In those cases, no DEGs could be predicted.

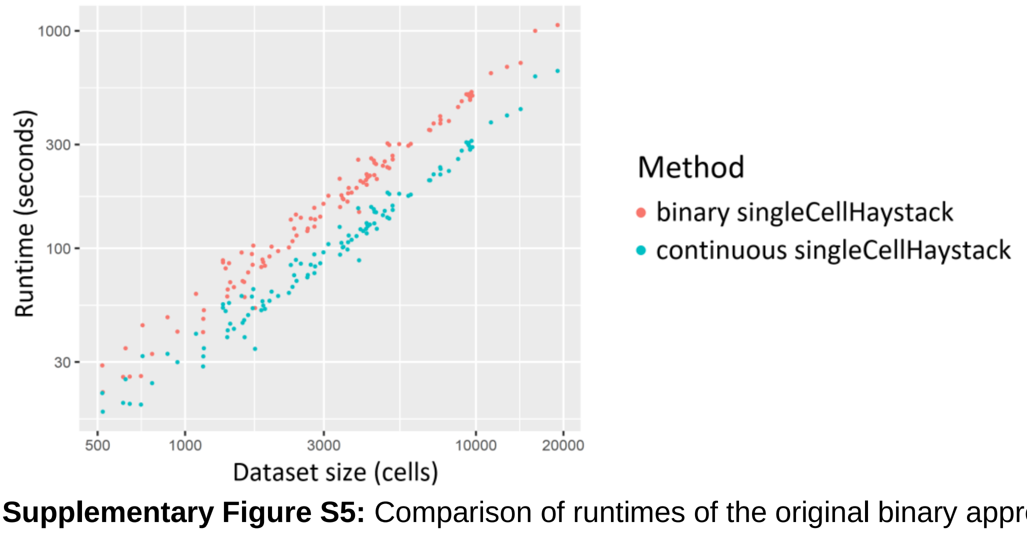

Supplementary Figure S5: Comparison of runtimes of the original binary approach (red) and the new continuous approach (blue) of singleCellHaystack on 119 scRNA-seq datasets of Tabula Muris and Mouse Cell Atlas.

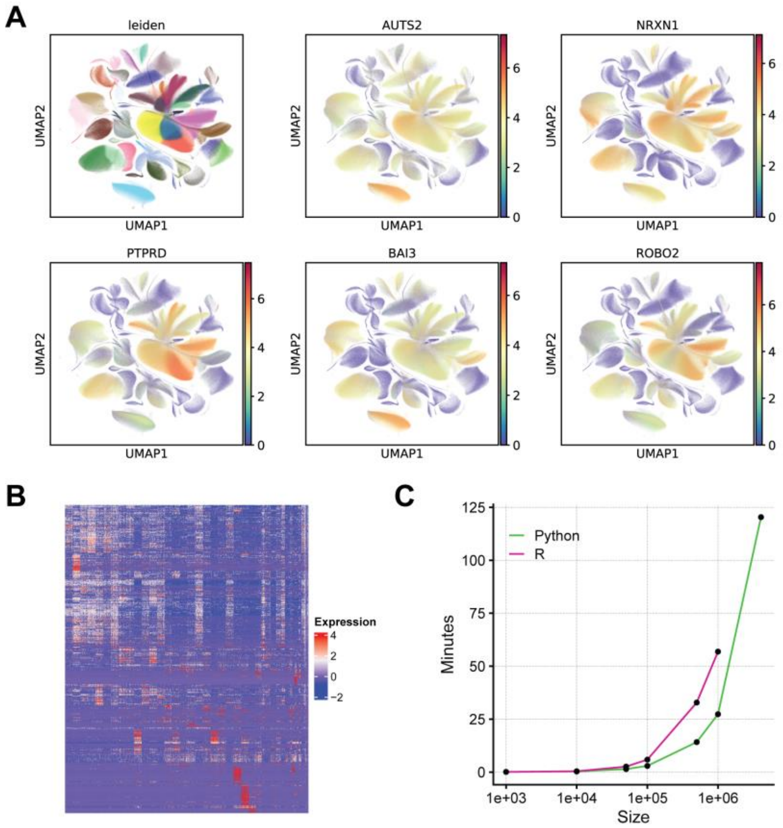

Supplementary Figure S6: Application to an atlas-level scRNA-seq dataset. **(A)** A UMAP plot of the 4.3 million cells in the Human Organogenesis Cell Atlas dataset, and a visualization of the expression of the 5 top-scoring genes identified with singleCellHaystack-py using coordinates of the first 50 PCs. **(B)** Heatmap of the top 500 genes predicted by singleCellHaystack using the 4.3 million cells. The heatmap shows their scaled expression in a subset of cells. **(C)** Running time for the R and Python versions of singleCellHaystack for different subsets of the full 4.3 million cell dataset.

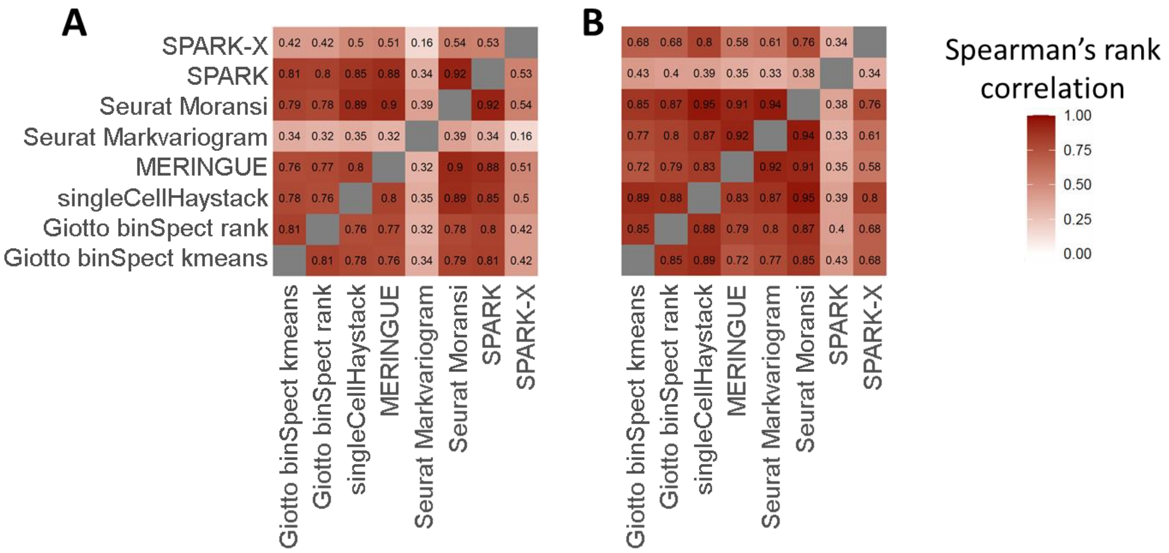

Supplementary Figure S7: Comparison of the results of SVG prediction methods. Heatmaps show the average Spearman's rank correlation between the p-values or scores returned by all methods, applied on the 1,000 HVGs of the 3 MERFISH datasets **(A)** and the 5 Visium datasets **(B)**. Values and colors reflect the Spearman's rank correlation values between each pair of methods.

(next page) **Supplementary Figure S8:** Comparison of results of singleCellHaystack and SPARK-X on four 10x Visium datasets. This figure supplements Figure 2B-D in the main paper. For each comparison, a scatterplot of p-values ($\log_{10}$) is shown on the left, and expression patterns of indicated SVGs are shown on the right. For each dataset, SVGs that are high-scoring according to one method but not the other are picked up. Datasets are posterior brain ("posterior2") **(A)**, anterior brain ("anterior1" and "anterior2") **(B-C)**, and kidney **(D)**.

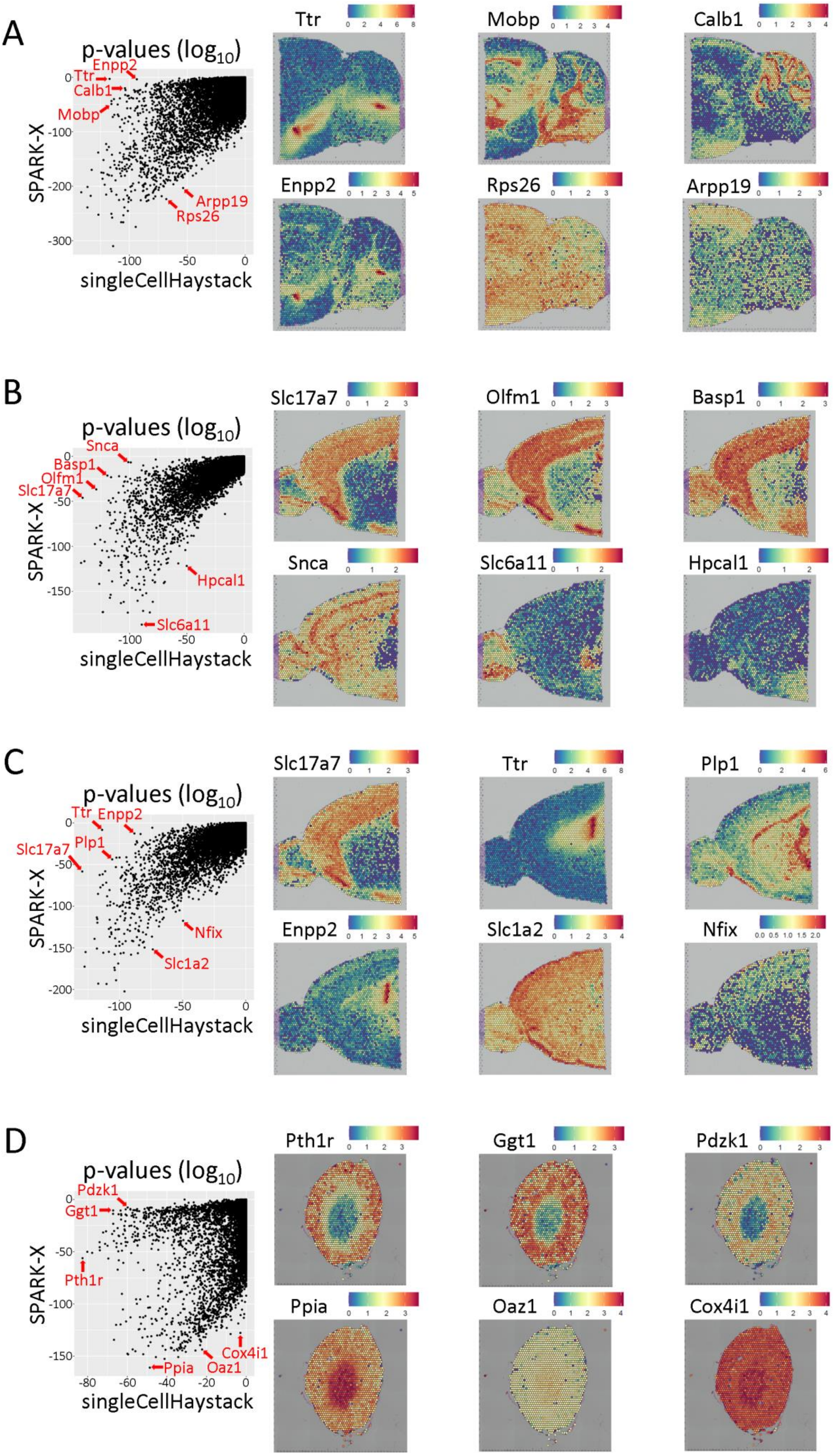
A
p-values (log10)
SPARK-X
singleCellHaystack
Ttr
Enpp2
Calb1
Mobp
Arpp19
Rps26
0
-100
-200
-300
-100
-50
0
Ttr
Mobp
Calb1
Enpp2
Rps26
Arpp19
B
p-values (log10)
SPARK-X
singleCellHaystack
Snca
Basp1
Olfm1
Slc17a7
Hpcal1
Slc6a11
0
-50
-100
-150
-100
-50
0
Slc17a7
Olfm1
Basp1
Snca
Slc6a11
Hpcal1
C
p-values (log10)
SPARK-X
singleCellHaystack
Ttr
Enpp2
Slc17a7
Plp1
Nfix
Slc1a2
0
-50
-100
-150
-200
-100
-50
0
Slc17a7
Ttr
Plp1
Enpp2
Slc1a2
Nfix
D
p-values (log10)
SPARK-X
singleCellHaystack
Pdzk1
Ggt1
Pth1r
Cox4i1
Ppia
Oaz1
0
-50
-100
-150
-80
-60
-40
-20
0
Pth1r
Ggt1
Pdzk1
Ppia
Oaz1
Cox4i1

(next page) **Supplementary Figure S9:** Comparison of results of singleCellHaystack and SPARK-X on four Slide-seqV2 datasets. This figure supplements Figure 2E in the main paper. For each comparison, a scatterplot of p-values (log_{10}) is shown on the left, and expression patterns of indicated SVGs are shown on the right. For each dataset, SVGs that are high-scoring according to one method but not the other are picked up. Datasets are from hippocampus **(A)**, embryo **(B)**, olfactory **(C)**, and cortex **(D)**.

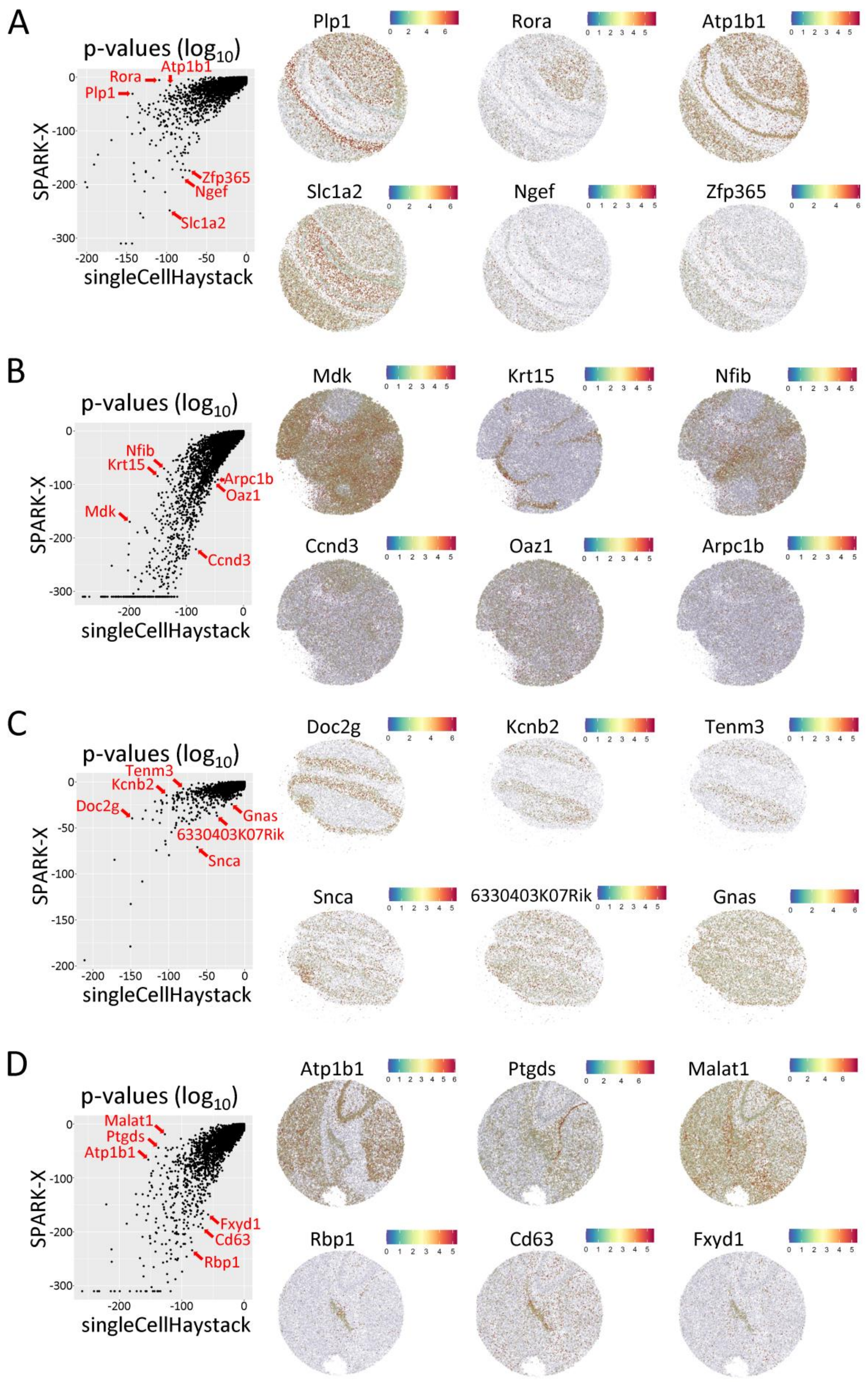
A
p-values (log10)
SPARK-X
singleCellHaystack
Plp1
Rora
Atp1b1
Zfp365
Ngef
Slc1a2
B
p-values (log10)
SPARK-X
singleCellHaystack
Nfib
Krt15
Arpc1b
Oaz1
Mdk
Ccnd3
C
p-values (log10)
SPARK-X
singleCellHaystack
Tenm3
Kcnb2
Doc2g
Gnas
6330403K07Rik
Snca
D
p-values (log10)
SPARK-X
singleCellHaystack
Malat1
Ptgds
Atp1b1
Fxyd1
Cd63
Rbp1

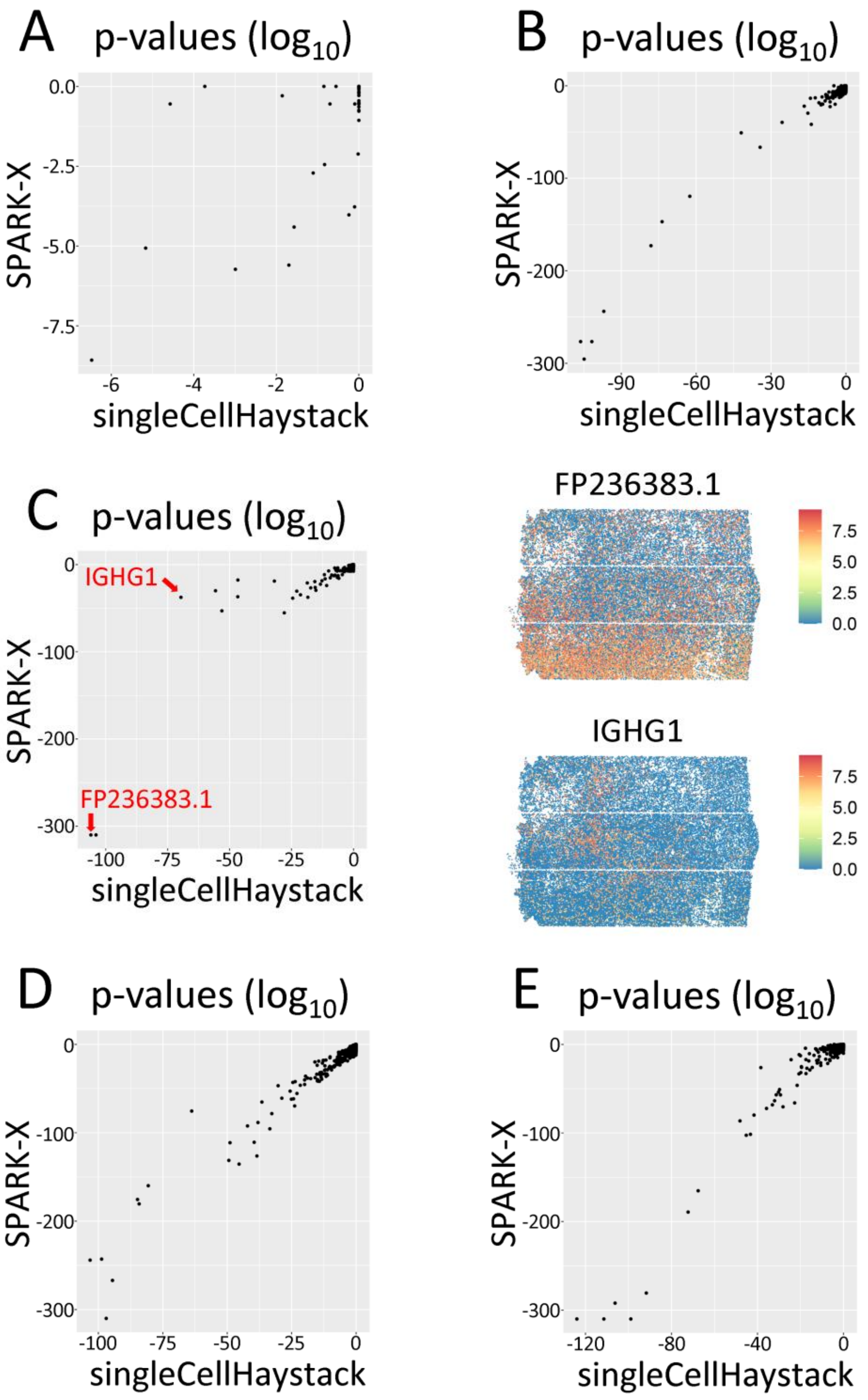

Supplementary Figure S10: Comparison of results of singleCellHaystack and SPARK-X on five HDST datasets. This figure supplements Figure 2D in the main paper. For each dataset, a scatterplot of p-values (log_{10}) is shown. In general, results returned by both methods are very consistent. In dataset vickovic_CN13_D2 **(A)** neither method found any SVGs. In datasets vickovic_CN21_C1 **(B)**, vickovic_CN21_D1 **(C)**, vickovic_CN21_E2 **(D)**, and vickovic_CN24_E1 **(E)**, high-scoring genes of one method were also high-scoring genes according to the other method. For vickovic_CN21_D1 **(C)**, the expression patterns of two genes are indicated as an illustration. Both are high-scoring according to both methods.

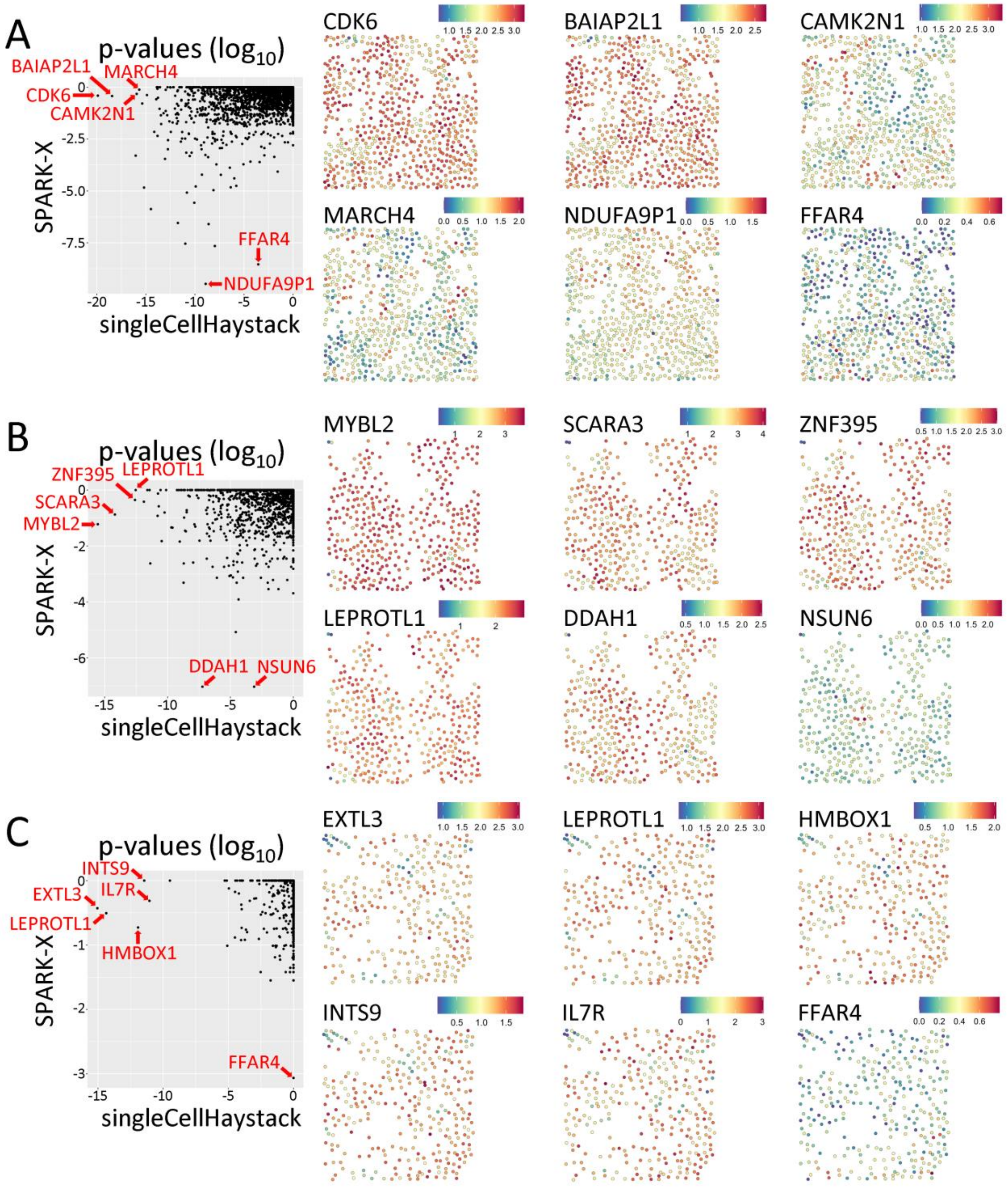

Supplementary Figure S11: Comparison of results of singleCellHaystack and SPARK-X on three MERFISH datasets. For each comparison, a scatterplot of p-values ($\log_{10}$) is shown on the left, and expression patterns of indicated SVGs are shown on the right. For each dataset, SVGs that are high-scoring according to one method but not the other are picked up. Datasets are Xia *et al.* B1 **(A)**, B2 **(B)** and B3 **(C)**.

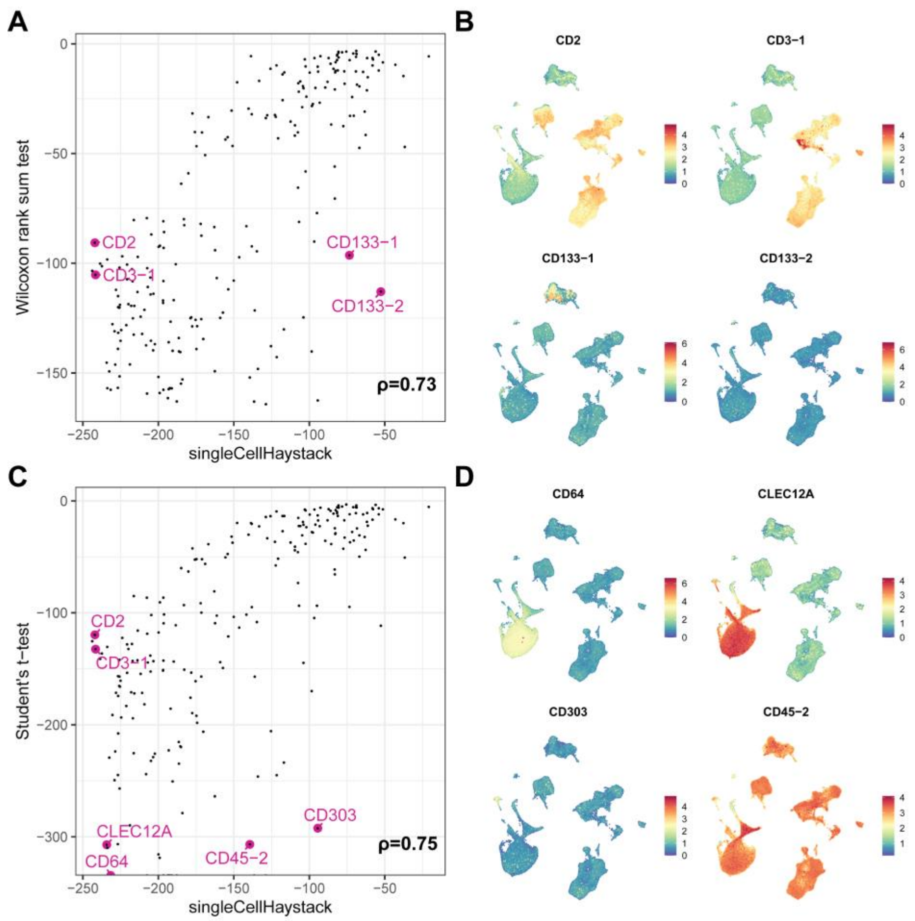

Supplementary Figure S12: Application of singleCellHaystack to a CITE-seq dataset. **(A-B)** Comparison between singleCellHaystack and the Wilcoxon rank sum test. Panel A shows the scatterplot of $\log_{10}$ p-values returned by singleCellHaystack (X-axis) and the Wilcoxon test (Y axis). Each point represents one protein in the CITE-seq dataset. Spearman's rank correlation coefficient (ρ) of the p-values is shown. Some proteins with different relative ranking in singleCellHaystack compared to the Wilcoxon test are highlighted, and their expression level patterns shown in panel B. **(C-D)** Comparison between singleCellHaystack and the Student's t-test. Panel C shows the scatterplot of $\log_{10}$ p-values returned by singleCellHaystack (X-axis) and the t-test (Y axis). Each point represents one protein in the CITE-seq dataset. Spearman's rank correlation coefficient (ρ) of the p-values is shown. Some proteins with different relative ranking in singleCellHaystack compared to the t-test are highlighted, as well as two proteins that were high-ranking according to both approaches (CD64 and CLEC12A). Their expression level patterns are shown in panel D. CD2 and CD3-1 are shown in panel B.

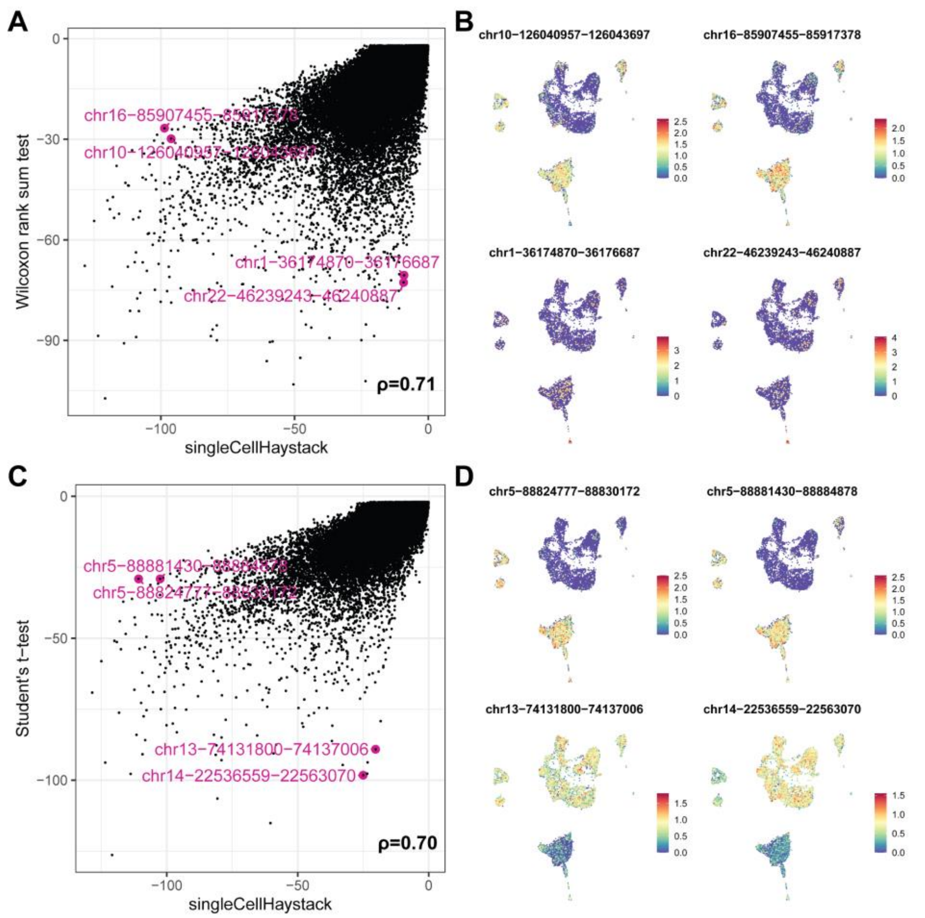

Supplementary Figure S13: Application of singleCellHaystack to a scATAC-seq dataset. **(A-B)** Comparison between singleCellHaystack and the Wilcoxon rank sum test. Panel A shows the scatterplot of $\log_{10}$ p-values returned by singleCellHaystack (X-axis) and the Wilcoxon test (Y axis). Each point represents one region in the scATAC-seq dataset. Spearman's rank correlation coefficient (ρ) of the p-values is shown. Some regions with different relative ranking in singleCellHaystack compared to the Wilcoxon test are highlighted, and their accessibility level patterns shown in panel B. **(C-D)** Comparison between singleCellHaystack and the Student's t-test. Panel C shows the scatterplot of $\log_{10}$ p-values returned by singleCellHaystack (X-axis) and the t-test (Y axis). Each point represents one region in the scATAC-seq dataset. Spearman's rank correlation coefficient (ρ) of the p-values is shown. Some regions with different relative ranking in singleCellHaystack compared to the t-test are highlighted, and their accessibility level patterns shown in panel D.

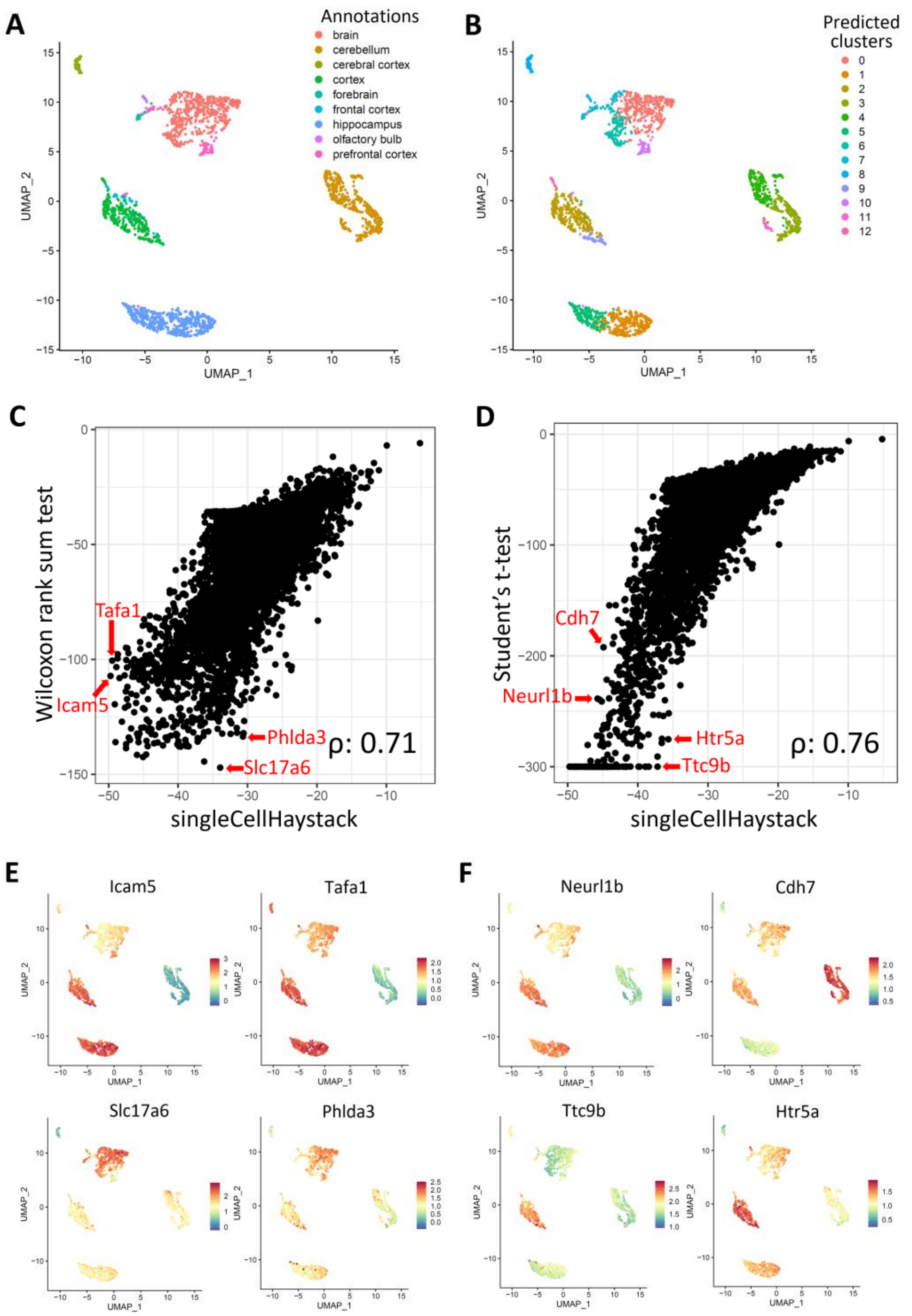

Supplementary Figure S14: Application of singleCellHaystack to a collection of bulk RNA-seq samples. **(A)** UMAP plot of the 1,958 RNA-seq samples obtained from various parts of the mouse brain. **(B)** The same UMAP plot showing the clusters estimated using the Seurat workflow (default resolution parameter). **(C-D)** Comparison between singleCellHaystack and the Wilcoxon rank sum test **(C)** and the Student's t-test **(D)**. The scatterplots show the $\log_{10}$ p-values returned by singleCellHaystack (X-axis) and the Wilcoxon test or t-test (Y axis). A pseudocount of 1e-300 was added to the p-values to avoid $\log_{10}(0)$ values. Each point represents one gene in the bulk RNA-seq dataset. Spearman's rank correlation coefficients (ρ) of the p-values are shown. Some genes with different relative ranking in singleCellHaystack compared to the Wilcoxon test or t-test are highlighted, and their expression patterns are shown in panels **(E-F)**.

SUPPLEMENTARY REFERENCES

1. Schaum, N. *et al.* Single-cell transcriptomics of 20 mouse organs creates a Tabula Muris. *Nature* **562**, 367–372 (2018).
2. Han, X. *et al.* Mapping the Mouse Cell Atlas by Microwell-Seq. *Cell* **172**, 1091–1097 (2018).
